# Frequency Patterns of T-Cell Exposed Amino Acid Motifs in Immunoglobulin Heavy Chain Peptides Presented by MHCs

**DOI:** 10.3389/fimmu.2014.00541

**Published:** 2014-10-28

**Authors:** Robert D. Bremel, E. Jane Homan

**Affiliations:** ^1^EigenBio LLC, Madison, WI, USA

**Keywords:** T-cell biology, regulatory T-cell, bioinformatics, B-cell:T-cell cooperation, polyspecificity, memory

## Abstract

Immunoglobulins are highly diverse protein sequences that are processed and presented to T-cells by B-cells and other antigen presenting cells. We examined a large dataset of immunoglobulin heavy chain variable regions (IGHV) to assess the diversity of T-cell exposed motifs (TCEMs). TCEM comprise those amino acids in a MHC-bound peptide, which face outwards, surrounded by the MHC histotope, and which engage the T-cell receptor. Within IGHV there is a distinct pattern of predicted MHC class II binding and a very high frequency of re-use of the TCEMs. The re-use frequency indicates that only a limited number of different cognate T-cells are required to engage many different clonal B-cells. The amino acids in each outward-facing TCEM are intercalated with the amino acids of inward-facing MHC groove-exposed motifs (GEM). Different GEM may have differing, allele-specific, MHC binding affinities. The intercalation of TCEM and GEM in a peptide allows for a vast combinatorial repertoire of epitopes, each eliciting a different response. Outcome of T-cell receptor binding is determined by overall signal strength, which is a function of the number of responding T-cells and the duration of engagement. Hence, the frequency of TCEM re-use appears to be an important determinant of whether a T-cell response is stimulatory or suppressive. The frequency distribution of TCEMs implies that somatic hypermutation is followed by T-cell clonal expansion that develops along repeated pathways. The observations of TCEM and GEM derived from immunoglobulins suggest a relatively simple, yet powerful, mechanism to correlate T-cell polyspecificity, through re-use of TCEMs, with a very high degree of specificity achieved by combination with a diversity of GEMs. The frequency profile of TCEMs also points to an economical mechanism for maintaining T-cell memory, recall, and self-discrimination based on an endogenously generated profile of motifs.

## Introduction

Immunoglobulin variable regions are a source of peptide diversity that is constantly being presented to the immune system and is continually changing as new epitope exposure occurs. B-cells are known to present MHC-bound peptides (pMHC) derived from the immunoglobulins, which they produce (1, 2). Indeed, immunoglobulin variable region peptides were among the first eluted from MHC class II (3). Other antigen presenting cells (APC), such as dendritic cells, take up immunoglobulins by binding their Fc receptors, alone or bound to exogenous antigens derivative peptides are then presented as pMHC.

**Figure 1:**
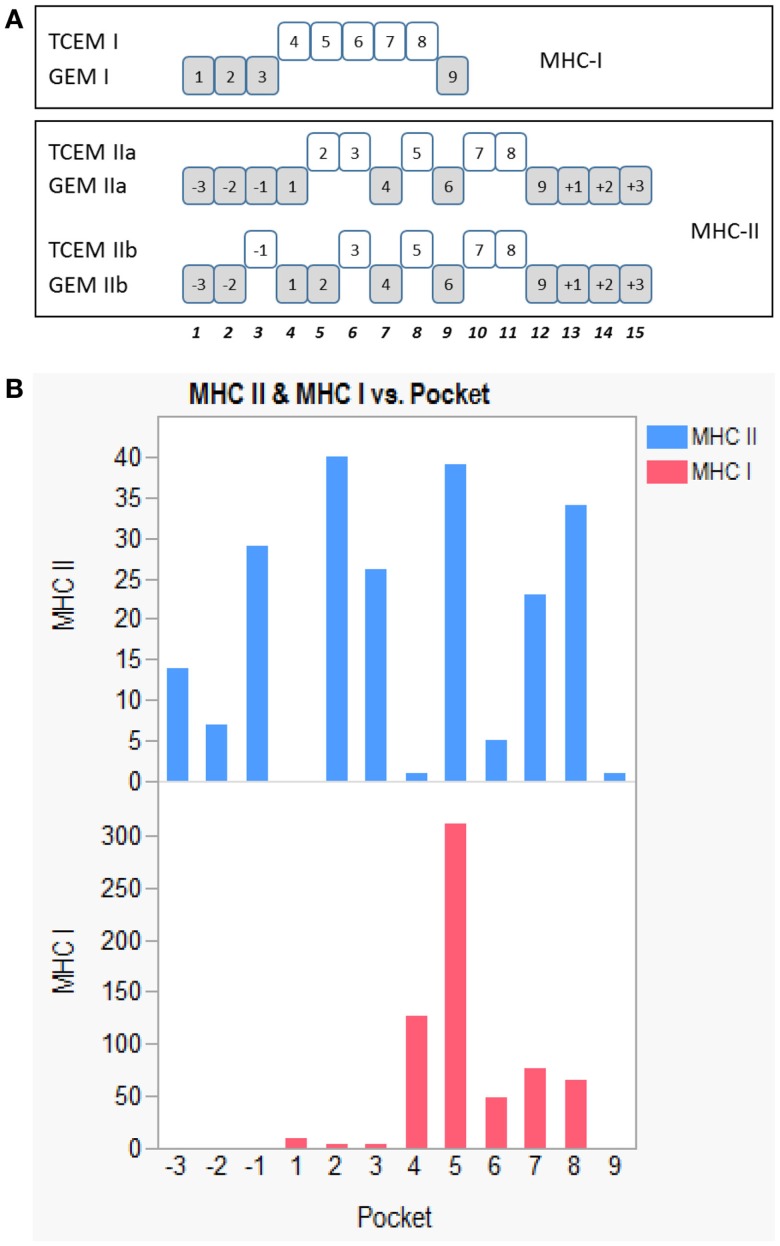
**Positions of the amino acids in TCEM and GEM registers**. **(A)** Non-continuous sets of amino acids in peptides were placed into categorical groups based on the structural analysis of amino acid contact points in pMHC:T-cell receptor complexes (4). For MHC-II, T-cell exposed motifs are related to pocket positions in a 15-mer, with a conventional 9-mer core flanked by three amino acids on each end. A 9-mer is shown for MHC-I. Two types of class II T-cell exposed motifs, TCEM IIa and TCEM IIb are considered. They comprise non-continuous amino acids and occupy the register positions shown. The obverse face of the 15-mer comprises the corresponding groove-exposed motifs, GEM IIA and GEM IIb, which are primarily involved in peptide binding to the MHC. The class I motif is a continuous series of amino acids occupying amino acid positions 4,5,6,7,8 in a conventional MHC class I pocket. The GEM I, amino acids 1,2,3,9 are primarily responsible for binding the peptide in the MHC-I. **(B)** Number of atomic contacts between the T-cell receptor and the peptide in the pMHC as tabulated by Rudolph et al. (5). X-axis shows MHC pocket positions, Y-axis the count of contacts.

Structural analysis of the T-cell receptor interaction with pMHC has shown that MHC-allele-specific binding affinity between the peptide and the MHC molecule is the function of a specific non-contiguous subset of amino acids, which face into the molecular groove (the groove exposed motif or GEM). A second subset of amino acids in the peptide, intercalated with those of the GEM, is exposed outwards to the T-cell receptor. Here, the T-cell exposed motif (TCEM) is recognized within the context of the atomic field of the outer histotope face of the allele-specific MHC molecule. The concept of two faces of the pMHC complex has been used to characterize host-microbe interaction (4). For MHC class I, the outward-facing amino acids making up the TCEM are identified as the central core of a 9-mer, comprising amino acids 4,5,6,7,8 (5, 6). In the case of the more open groove of MHC class II molecules, two possible binding registers allow for TCEMs comprising either amino acids 2,3,5,7,8 or -1,3,5,7,8, where the numbering is N-C and is based on the central 9-mer core of a 15-mer. We identify these registers as TCEM IIa and TCEM IIb, respectively. The numbering of the amino acid registers in the motifs are shown in Figure 1.

The antigenicity of biotherapeutic antibodies is thought to be largely dependent on the helper CD4+ T-cell response to such molecules (7). To understand the patterns of processing and presentation by APC of peptides from antibody molecules, we decided to examine a large dataset of naturally occurring immunoglobulin heavy chain variable region (IGHV) sequences. These analyses revealed distinct regional patterns of predicted high affinity pMHC binding within the IGHV. In addition, the TCEMs in the variable regions of both germline-origin and somatic hypermutated (SHM) sequences exhibit distinct frequency patterns of re-use. TCEM of both germline and SHM-origin found at high frequencies are each associated with a range of GEM, thereby conferring different MHC-allele binding patterns when the peptide is presented in the pMHC complex. Characteristic patterns of TCEM found in heavy chain constant regions are distinct from those of IGHV. Frequency patterns of TCEM re-use in combination with variable MHC-allele-specific binding patterns provide a framework for understanding immunological memory and self-discrimination.

## Results

We assembled and curated several non-redundant databases consisting of non-class-defined IGHV (approximately 40,000 sequences, “the 40K set”), class-defined IGHV (2,834 sequences), IGHV germline families (161 sequences), and the human proteome (81,000 proteins, excluding immunoglobulins). For each of the database protein sequences, from every sequential 9-mer and 15-mer sub-peptide, indexed by single amino acid displacement, we extracted the TCEM and GEM motifs. We further computed the predicted binding affinity for human MHC class I (for 20 MHC class IA and 17 MHC class IB alleles) and MHC class II (16 DR, 6DP, and 6DQ alleles) and the probability of excision of each peptide by cathepsin B, L, and S (8).

### Patterns of predicted MHC binding affinity

Sequences from each of the IGHV germline families exhibit distinct, but similar, patterns of predicted MHC binding affinity. To simplify the description of the multi-dimensional patterns in this aspect of our analysis, we will refer to sequences of IGHV3 germline-origin (9), which is the most prominent family and comprised approximately 56% of the 40K dataset. Graphics of the other IGHV families are found in Figure S1 in Supplementary Material.

**Figure 2:**
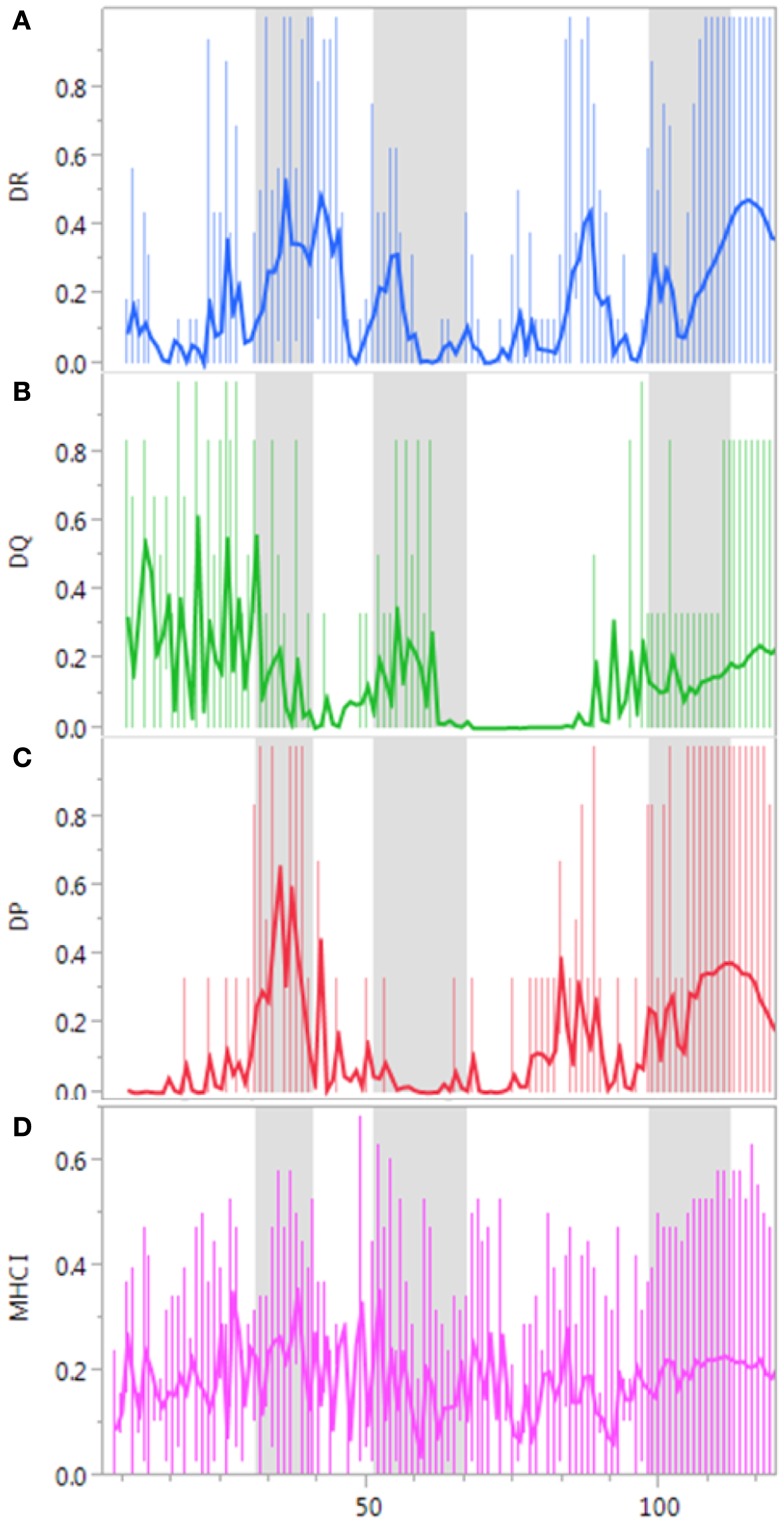
**Predicted MHC binding affinity and frequency distribution of TCEM repeats**. **(A–D)** The predicted MHC binding affinity of all sequential peptides derived from a subset of 10,000 IGHV3-origin variable regions. A: DR, B: DP, C: DQ alleles MHC class II (15-mers), and D: MHC class I A and B alleles (9-mers). The binding affinity is expressed as the fraction of alleles binding the peptide centered at the indicated position, where the binding affinity of the peptide to an allele is predicted more than 1 σ below the mean for that allele within the particular parent protein. The solid line represents the average and the extensions show the 10 and 90% points. Thus, a predicted affinity as seen for DR centered at aa 38 of 0.62 indicates that on average 10 of the 16 DR alleles for which predictions are made are predicted to bind this peptide with an affinity in excess of 1 σ below the mean. As shown by the extensions some peptides are bound by essentially all MHC class II alleles evaluated. The gray shaded background indicates the approximate location of the three CDRs. Corresponding plots for other IGHV families are shown in Figure S1 in Supplementary Material and for IGHV3 germline in Figure S2 in Supplementary Material.

The pattern of MHC class II binding in the IGHV3 molecule subset (heavy chain class undefined) is shown in Figures 2A–C. There are several regions where peptides generated by SHM have predicted high affinities for most MHC class II alleles. In other regions, the peptides have uniformly low predicted binding affinities. Patterns of predicted binding affinities of DP and DR alleles are similar, but differ from the DQ alleles. In particular, the DQ alleles have a noticeable binding preference for peptides in framework (FW) region 1. The patterns of predicted pMHC binding affinity before SHM (i.e., germline-origin motifs), and after SHM are similar, except that CDR3 is absent in germline (Figure S2 in Supplementary Material). This indicates a retention of binding characteristics of the GEMs as novel TCEM are produced by SHM. In contrast to MHC class II, the predicted binding pattern of MHC class I alleles to IGHV peptides shows no distinct regions of higher affinity (Figure 2D). Cleavage by cathepsins and other endosomal peptidases determines excision to enable presentation of peptides from the IGHV in the pMHC. SHM changes the distribution of predicted cleavage sites in each IGHV, although certain regions remain more resistant to cleavage (Figure S3 in Supplementary Material).

### Frequency distribution of unique TCEM in germline and somatic hypermutated variable regions

We extracted and classified each of the TCEM I, TCEM IIa, and TCEM IIb pentamers from the peptides in the database of 161 germline IGHV and from the approximately 4.4 × 10^6^ individual peptides in the 40K set of SHM IGHV. For any MHC binding peptide, the theoretical maximum of possible unique pentamer amino acid combinations for each TCEM class is 3.2 × 10^6^ (20^5^). However, considering all peptide positions within the 40K set, only approximately 275,000 unique motif sequences were found for each TCEM register (TCEM I = 275,176; IIa = 273,017; IIb = 276,034). Thus, TCEM motifs found in the IGHV are each used many times over in the dataset. Even considering that some germline sequences are retained, only a small fraction of the possible diversity of TCEMs are found. Furthermore, in 32% of instances a unique TCEM IIa was co-located with a unique TCEM IIb, so a hexamer may comprise motifs, which engage two different unique T-cell populations.

**Figure 3:**
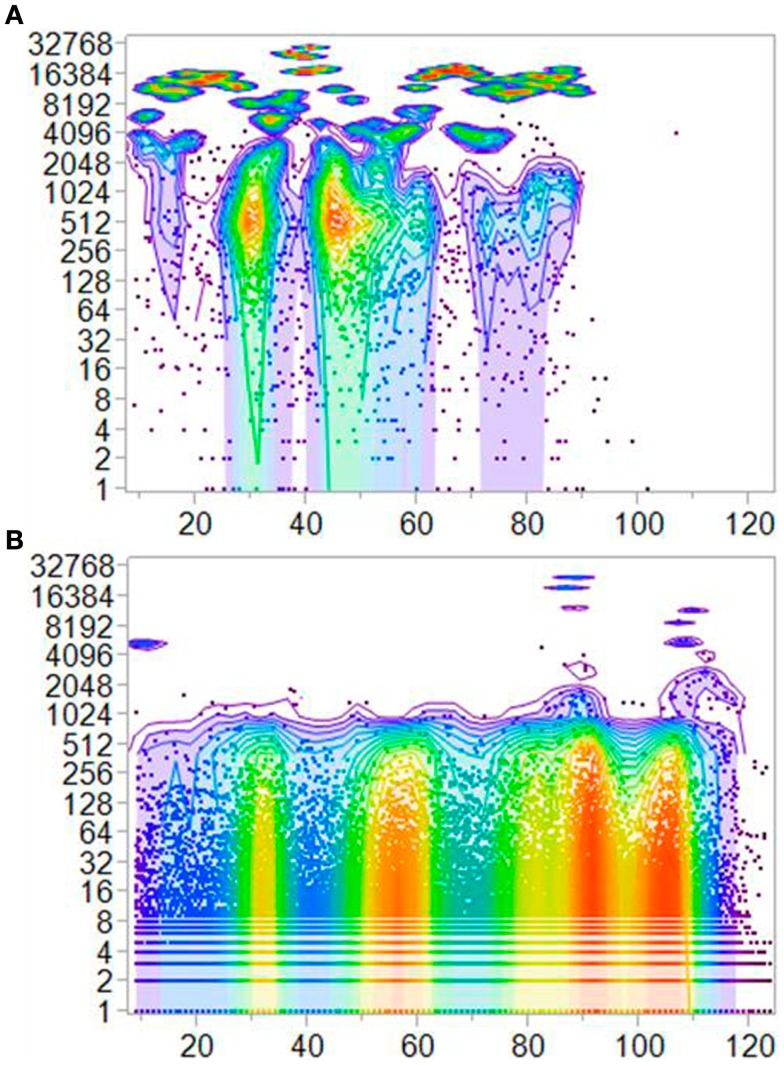
**Shows the frequency of occurrence (log_2_) of unique TCEM IIa T-cell exposed motifs in the IGHV region of the IGHV3-origin sequences (22,458 IGHV, 2.2 × 10^6^ motifs)**. The number of unique motifs at each amino acid position is plotted at the center of the motif in the IGHV. **(A)** Motifs identical to those found in germline sequences. **(B)** Motifs produced by somatic hypermutation and not found in the germlines. The coloration is created by a bivariate clustering algorithm that produces a spectral distribution on 5-percentile boundaries. Corresponding plots for TCEM IIb and TCEM I are shown in Figure S4 in Supplementary Material and for other IGHV families are shown in Figure S5 in Supplementary Material.

Figure 3 shows the frequency distribution for IGHV3 TCEM IIa. The corresponding distributions for other TCEM registers are provided in Figure S4 in Supplementary Material. A quantile density contouring algorithm was used to generate color gradation contours to show the regions with most repetition of TCEM within the databases. The complementarity determining regions (CDRs) are clearly visible as the regions of highest repetition.

**Figure 4:**
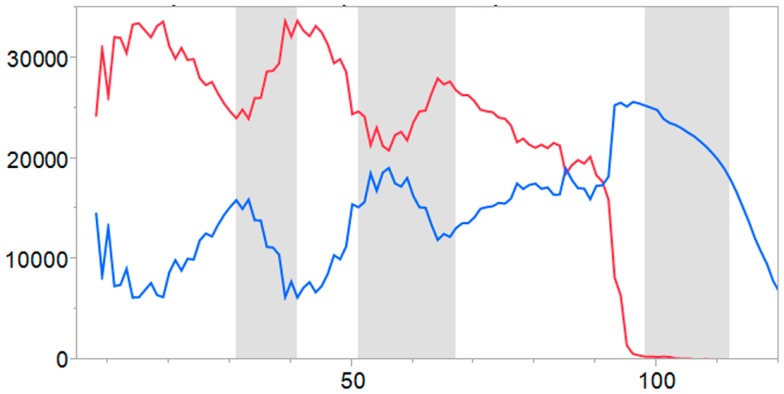
**The frequency of unique motifs as a function of amino acid centered location for the entire 40K dataset**. The red line plots the number of unique germline-origin motifs and the blue line the unique motifs generated by SHM.

For each of the TCEM registers, 60–63 germline motifs are found un-mutated in approximately 25% of the 40K set of molecules. Within the SHM sequences there are TCEMs resulting from hypermutation, which also show a high degree of repetition. In both cases, the repeated TCEM are affiliated with a range of SHM-generated GEMs, leading to wide variation in predicted pMHC affinity. Figure 4 shows that the frequency pattern of unique motifs in SHM is counter to that of germline and progressively increases through the IGHV with most diversity at the N-terminal side of each of the CDRs. Taken together with the data in Figures 2A–C, it is clear that regions of high motif diversity are also regions in which many alleles are found to have predicted high affinity pMHC binding. As the SHM mechanism is stochastic, hypermutation of GEMs and TCEMs are independent, and thus a wide range of pMHC affinities may coexist with each conserved TCEM. Furthermore, the affinity of each GEM differs based on host immunogenetics. Corresponding plots of GEM frequency show similar repetitive patterns of these non-continuous motifs, uncorrelated with TCEMs (not shown).

### Frequency distribution classification

Examination of the patterns in Figures 3 and 4 indicates a gradation in the pattern of TCEM re-use. We therefore devised a numerical frequency classification (FC) system for the motifs, using reciprocal base-2 logarithmic categories to define a motif FC system. Hence, FC2 indicates a motif found in 1/4 (1/2^2^) clonal B-cell immunoglobulin products, FC10 is 1/1024 (1/2^10^). Conveniently, this also provides a potential T-cell stimulation metric in which, at constant pMHC affinity, each increment in FC represents a halving of the potential frequency of T-cell: APC encounters. It also provides a characteristic T-cell-relevant metric that can be applied to TCEM found in other proteins.

**Figure 5:**
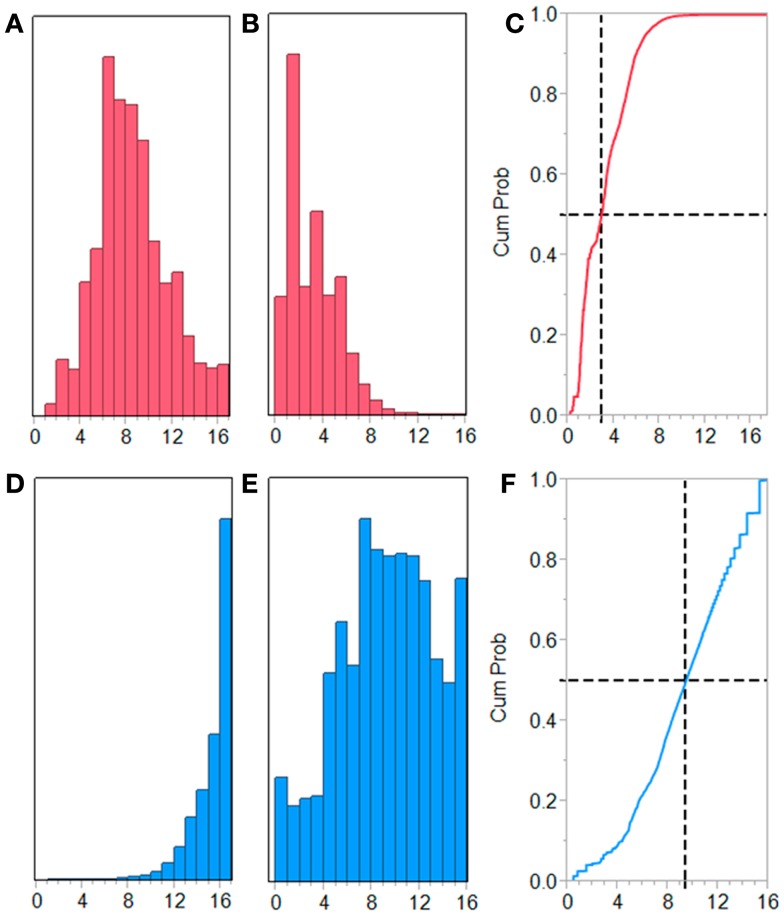
**Frequency distributions of unique T-cell exposed motifs in IGHV3**. Frequency distributions of unique TCEM IIa motifs found in 22,458 IGHV3-origin sequences, comprising a total of 2.2 × 10^6^ possible motifs. **(A–C)** Germline-origin motifs and **(D–F)** SHM-origin motifs. **(A)** and **(D)** histogram of frequency of occurrence (histogram bins as −log_2_). **(B)** and **(E)** frequency weighted histogram. **(C)** and **(F)** cumulative distribution frequency of B and E. The dashed lines depict the frequency distribution midpoint.

Each IGHV germline-origin family exhibits minor differences in motif repetition patterns, likely owing to differences in frequency of cytidine-containing codons in the underlying nucleotide sequences undergoing SHM (Figure S5 in Supplementary Material). Figure 5 shows the frequency distribution patterns for IGHV3. The underlying data are provided in Table S1 in Supplementary Material. Here, we see that 80% of the germline-origin motifs are found in the commonest frequency categories, FC1, 2, and 3. In contrast, for SHM-origin motifs, 50% of the cumulative TCEM IIa motifs occur in FC1-10, with most occurring between FC5 and FC10. This corresponds to repetition in about 1/32 to 1/1000 clonal-origin cells. Hence, between 3 and 0.1% of all B-cells share somatically hypermutated sequences that may be MHC bound and are exposed to T-cells. In addition, SHM sequences in the 40K set have a high count of approximately 140,000 FC16 motifs. These are motifs that are each found only once in the 40K set. When considered on a per molecule basis this amounts to between three and four unique motifs per IGHV, all others are recurrent (Figure S1 in Supplementary Material).

Only approximately 40,000 unique IGHV sequences could be assembled from Genbank. Increasing the size of the database might add more counts to each frequency class but will not change the distribution or alter the fact that >90% unique motifs occur in FC1–15. This is confirmed by analysis of the non-redundant immunoglobulin class-defined subsets (below).

In summary, these results indicate that multiple, high affinity, repeated TCEM are found in a relatively high proportion of B-cell clonal lines and antibodies from them. It also shows that rarer motifs occur more often in SHM sequences than in sequences of germline-origin and tend to be affiliated with higher affinity GEMs.

### Recurrent patterns of TCEM and GEM

The peptide which eventually becomes bound in an MHC and thus exposes a TCEM is flanked by peptides, which determine the probability of endosomal peptidase excision. Such peptidases, including the three principal cathepsins we predict, recognize an octomer spanning four amino acids either side of the cleavage site (the cleavage site octomer or CSO). Overall therefore the selection of a T-cell exposed pentamer, that has been excised and bound in a pMHC, depends on a peptide that spans 23 amino acids (4 + 15 + 4). In an immunoglobulin variable region, a 23-mer extends across boundaries of FW and CDR. We will select one peptide position to illustrate how: (i) a wide variety of frequencies are encountered at any one position in IGHV; (ii) the same peptide can encompass both germline-origin and SHM-origin sub-sequences of TCEMs with each of these sub-sequences occurring at different frequencies, and (iii) a particular TCEM can be affiliated with a wide range of GEMs, conferring a range of different affinities for different HLA alleles.

**Figure 6:**
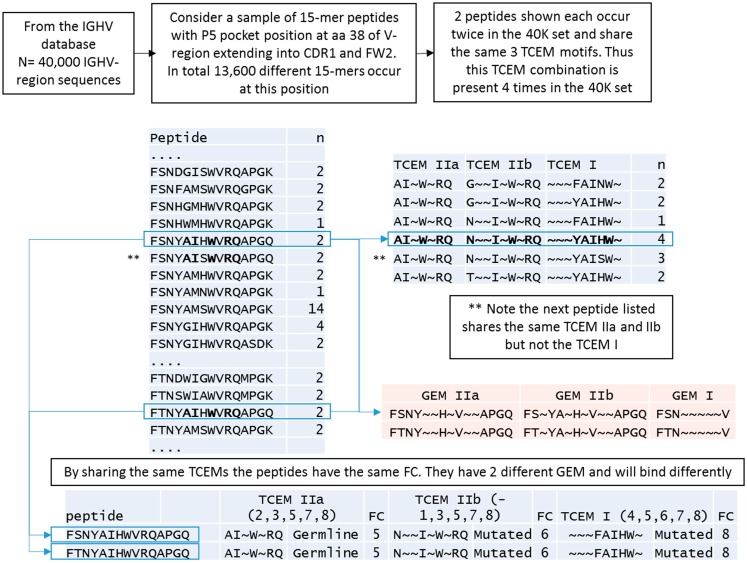
**Examples of the interrelationships of TCEM and GEM motifs**. A subset of peptides centered at IGHV position 38 is shown to illustrate the cryptographic overlay of TCEM and their associated GEM. The number of times that particular peptide or motif group occur in the database is shown as “n.” In the lower panel FC represents the −log_2_ frequency classification of the particular motif and germline and mutated indicate the origin of the motifs.

**Figure 7:**
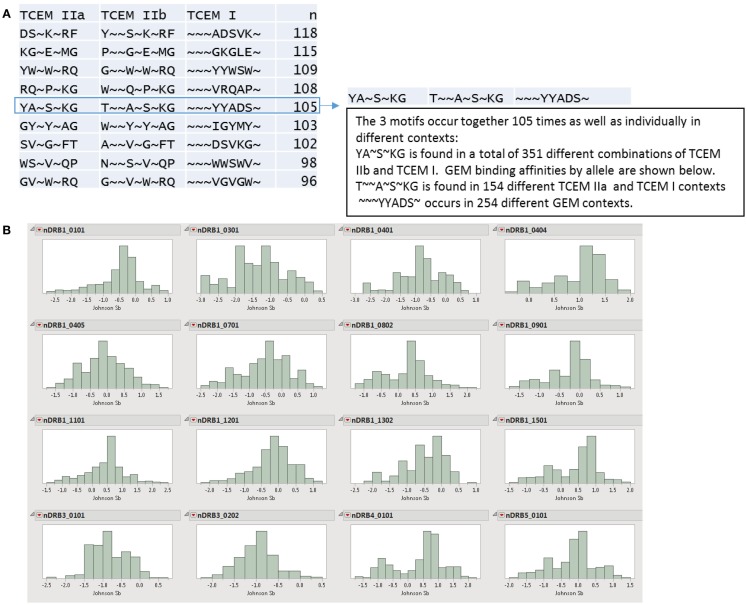
**Allele-specific binding profiles of GEM motifs**. **(A)** Examples of combinations of TCEM that occur together approximately 100 times. There are many different GEMS associated with each that have a range of affinities. **(B)** DRB allele affinity distribution histograms of the 351 peptides where TCEM IIa YA~S~KG is found. This shows the role of independent mutation of the GEM on binding affinity. The affinities of each allele within each IGHV are standardized to zero mean and unit variance with a Johnson Sb distribution algorithm and the *x*-axis is in standard deviation units with the mean being zero. A high bar in the histogram for nDRB1*01:01 between −0.25 and −0.50 indicates that a large fraction of the total are found with affinities in this range. The majority of the peptides with this motif are below the mean affinity for that particular allele in all of the 351 peptides reflecting the fact that the CDR regions tend to be regions with high affinity binding. The long tail toward increasingly negative numbers indicates that some peptides with this particular motif have very high overall affinities (−2σ), among the highest in the particular IGHV. For some alleles, these peptides have a significantly lower relative affinity and in some cases such as DRB1*08:01 appear to be bimodal. Non-standardized log_e_ distributions of the same data are shown in Figure S6 in Supplementary Material.

Within our dataset there are 40,000 peptides centered at position 38 of the IGHV. These include both SHM and germline-origin TCEM motifs with a wide range of frequency categories. Peptides in this region of the IGHV tend to have a high probability of excision by each of the three cathepsins for which we make predictions. Thus they would be expected to be processed in both the B-cells, which do not express cathepsin L (10) as well as other APC that express all three cathepsins. Figure 6 shows a small subset of the 40K peptides at this position, selecting as an example of convenience, a peptide FSNYAIHWVRQAPGQ, which occurs twice in the database. A second peptide, differing by a single amino acid, T vs. S at position 2, shares all three TCEM. While shared in this peptide, each of the three TCEMs also occur individually many other times in other combinations. The AI~W~RQ motif is found in the germline at FC 5; it is found in 1 in 32 B-cell clonotypes. The other MHC class II motif, N~I~W~RQ is the result of SHM and is found in 1 in 64 B-cell clonotypes. The MHC class I motif FAIHW, is also the result of SHM and is found 1 in 256 clonotypes. Figure 6 also shows that the GEMs associated with the two peptides are different in every register. Figure 7 illustrates how a range of affinities are associated with a specific set of TCEMs. For this purpose, we selected a different peptide, also centered at position 38, which has a set of TCEMs that occur in combination with each other 105 times (Figure 7A). Each of the different motifs is also found in combination with other motifs and at different frequencies. The histograms in Figure 7B are the predicted within-protein standardized DR affinities for the 351 15-mers that contain the MHC class II TCEM IIa YA~S~KG. Corresponding non-standardized distributions of the actual log_e_ IC_50_ are shown in Figure S6 in Supplementary Material. The YA~S~KG motif is found peptides with a very wide range of affinities resulting from SHM of the GEM regions. While it cannot be identified in the histograms, the YA~S~KG-containing peptides with any given GEM will have a high affinity in one set of alleles but a different affinity for other alleles. Thus, even though two TCEM may have the same frequency of occurrence, they will generate different T-cell responses because different dwell times as pMHC result from the differences in affinities among the HLA alleles.

**Figure 8:**
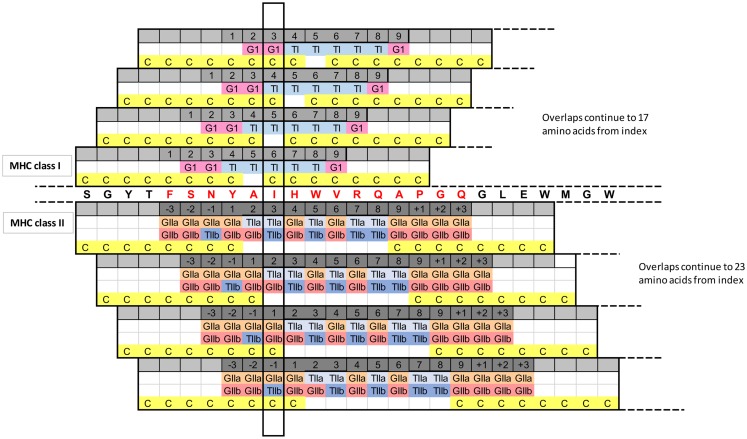
**Overlapping recognition frames indicate the impact of a single amino acid mutation**. A single peptide is shown in the center, from the same example as Figure 6. The TCEM, GEM, and cathepsin cleavage site octomer recognition frames are shown for this and for the overlapping peptides starting 1, 2, and 3 index positions downstream. The vertical box shows how a single amino acid mutation at this position would impact many different recognition functions across the overlapping peptides. Only four iterations are shown; the overlap actually extends 17 amino acids (class I) or 23 amino acids (class II), assuming the default size of binding peptides of 9-mer and 15-mer. This pattern is repeated for each index position in a protein (i.e., approximately 130 times for a variable region). GI, GIIa, and GIIb indicate amino acids that are part of the GEM and contribute to binding; TI, TIIa, and TIIb indicate the TCEM amino acids. C indicates an amino acid, which is part of the cathepsin cleavage site octomer. The peptide in this example was from gi 122892104.

Referring again to our selected peptide in Figure 6 centered at position 38, the 15-mer comprises the multiple overlaid TCEM I and IIa and IIb registers and the surrounding and intercalated GEM amino acids and has peptidase CSOs extending beyond both the C-terminal and N-terminal sides of 15-mer. Mutations anywhere in nearly 20% of the entire length of the variable region will therefore impact the behavior of our selected 23-mer peptide. If we now consider the peptide centered one amino acid along, at peptide position 39, the amino acid which in our original peptide was the innermost position in the CSO flank is now in the GEM of our new peptide, and an amino acid, which was in the GEM is now part of the TCEM. Every amino acid has a role in 22 more overlaid peptide spans, and every mutation in one peptide has an impact on the overlapping peptides. Figure 8 illustrates this complexity.

### Class-defined immunoglobulins

**Figure 9:**
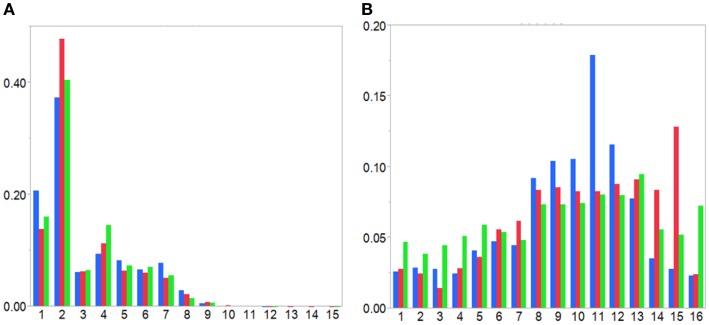
**Distributions of T-cell exposed motif by frequency class for immunoglobulin class-defined IGHV**. Histograms of distributions of TCEM IIa by frequency class for immunoglobulin class-defined IGHV. **(A)** Germline-origin sequences and **(B)** SHM-origin motifs. IgG: red, IgM: green, and IgE: blue. A frequency classification for each motif was created by binning motifs based on the frequency in the 40K database on a −log_2_ scale. For the Ig class-defined datasets shown here the TCEMs in each successive peptide indexed by a single amino acid in the IGHV were then assigned to a frequency class from the main database.

Analysis of the non-redundant class-defined subsets shows that mutated IgG, IgE, and IgM have different patterns of distribution across frequency class (Figure 9). As expected, because IgG has undergone a greater degree of SHM, more rare (higher FC) TCEM are found in IgG than in IgM. IgE has few rare motifs but many more FC8–13. While different from IgG, it is unclear whether this is a characteristic of IgE generally or might be biased by inclusion in the database of a large number of samples from a study of asthmatic children (11–13). Analysis of the TCEM compositions of the three class-defined sets showed that all of the motifs in the IgG subset were present in the separate 40K database. In the IgM and IgE data sets, <0.03% motifs were absent from the 40K database. Thus, while the class-defined sets are relatively small, the TCEM patterns in the 40K set are representative of the different immunoglobulin classes. Corresponding frequency distributions were generated for TCEM I. These show similar frequency distributions as for TCEM IIs and the underlying data are included in Table S1 in Supplementary Material. However, as Figure 2 shows, there is no association of repetitive TCEM I with high affinity MHC binding in specific regions of the sequences.

### TCEM motifs in heavy chain constant regions

Peptides with T-cell suppressive activity have been reported in heavy chain constant regions and thus it is of interest to examine the types of TCEMs found in the Fc region of immunoglobulins (14, 15). We examined the three registers of TCEM found in sequences of the heavy chain constant [immunoglobulin heavy chain constant regions (IGHC)] region of IgA, IgD, IgE, IgM, and IgG1, IgG2, IgG3, and IgG4 and evaluated the extent of overlap with TCEMs found in IGHV. The VDJ region was not included in this analysis because of its variable length; only those motifs after the cysteine at amino acid position 25–27 of the constant region were included. TCEM in the IGHC differ from the IGHV. Only a small proportion of the motifs found in IGHC were also found in the IGHV 40K set (Table S2 in Supplementary Material). In IgG1, IgG2, and IgG4 only 1–4.25% of IGHC TCEM were repeated in IGHV. In contrast, IgE showed 9–10% of re-use of IGHV motifs in IGHC. IgG3 was an outlier with over 15% of IGHC motifs matching IGHV motifs; these matches were located in the extended hinge region of IgG3. We also predicted the binding affinities for all substituent peptides in the constant regions. Each of the constant regions have potentially excised peptides with predicted high affinities for a number of different MHC alleles. As the GEM and TCEM context of any peptides derived from the constant region should indeed be constant, they appear in every sequence and thus are classified as FC0 (2°).

### Comparison of TCEM usage in IGHV and in the human proteome

The UniProt human proteome database was curated to remove immunoglobulin sequences. The dataset includes all isoforms of gene products and comprises approximately 81,000 proteins, an average redundancy of about fourfold a single proteome. In aggregate, the proteome database has about twice as many proteins as the IGHV database, but comprises proteins of larger average size. The proteome database has a total of 33 × 10^6^ 15-mer peptides, compared to approximately 4.4 × 10^6^ in the IGHV database. When processed as for the IGHV database, the proteome produced about 2.42 × 10^6^ unique motifs for each TCEM register (I, IIa, and IIb). Thus about 75% of the 3.2 × 10^6^ (20^5^) theoretical possible TCEMs are used. While different isoforms of proteins in this set may vary slightly in TCEM content, unlike immunoglobulins the peptide content of the rest of the proteome is not undergoing constant change in response to the internal and external environment and is not consistently presented by professional APCs. The intersection between the proteome and IGHV TCEM datasets show that about 85.5% of TCEMs found in the IGHV match those found in the proteome set (Figure S6 in Supplementary Material). When the FC classification derived from immunoglobulins was applied to the proteome set, the mean frequency was at FC12, showing that there is a high degree of motif recurrence shared between IGHV and the proteome. Thus, we conclude that the IGHV has characteristics that makes it a motif self-reference set for the entire proteome.

## Discussion

Immunoglobulin variable regions are a source of tremendous peptide diversity that undergoes constant innovation in response to new epitopes. APC, including B-cells, continually process and present immunoglobulin-derived peptides as pMHC, just as they do other peptides from self and non-self proteins. In the case of B-cells, the immunoglobulin variable region peptides are of endogenous origin, whereas dendritic cells and macrophages acquire immunoglobulins through Fc-mediated uptake. Processing and presentation of peptides derived from immunoglobulins likely exceeds that from the rest of the human proteome, both in diversity and continuity, and thus is likely to play an important part in shaping T-cell immunity. We show a distinct pattern of distribution within IGHV of peptides, which have high predicted binding affinity for MHC class II molecules.

While the variable regions of immunoglobulin molecules are generally considered to have very high variation, we show there is a surprisingly high frequency and consistent pattern of re-use of TCEMs in peptides derived from variable regions. This is the case across all three TCEM registers examined. Sequences conserved from germline and sequences resulting from SHM differ in their frequency of TCEM re-use. Germline-origin motifs are found predominantly in FC 1–3 (appearing in one in two to one in eight clonal B-cell immunoglobulin products). In contrast, half of the SHM-origin TCEM repertoire is re-used at least once in every 1024 B-cell clonal variable region products (FC1-10) and some SHM-origin motifs are present in as many as half of all antibodies (FC1). Rare TCEMs (FC16), found only once in our 40K database, occur only in mutated sequences. Overall the ratio of germline to SHM TCEMs is approximately 60:46. This ratio represents the overall proportion of motifs in the final molecule that are identical to that found in the germline vs. those which result from SHM. There are only minor differences between the TCEM frequency distributions in IGHV derived from different classes of immunoglobulin.

An adult human is estimated to carry 3–9 × 10^6^ unique CDR3 (16), so our composite dataset of 40,000 unique IGHV is equivalent in size and diversity to about 1% of the B-cell clonotype population in an individual. Deposited in Genbank by multiple investigators, the set is a representative sample of the possible diversity. Given the frequency distribution of TCEM, increasing the database size would still lead to >50% of motifs being in FC1–10, with an expected increase in the number of singleton (FC16 and above) motifs. The FC16 motifs in the 40K dataset comprise approximately 140,000 motifs, which each occur only once. Each IGHV clonotype we examined thus contained only 3–4 unique TCEM. These are found in positions throughout the IGHV but predominantly in the CDRs. The TCEM found in the separate class-defined subsets were virtually all in the larger database, validating the patterns for the B-cell repertoire in general.

The constant region utilizes a different vocabulary of TCEM; there is only a very small number of motifs in the constant regions that are shared with the variable regions. Their invariance suggests that T-cells, which recognize constant region FC0 TCEM, when these are affiliated with high affinity GEMs, most likely mediate negative selection and/or deletion of thymocytes (17). Further, the diversity of TCEM use in the constant regions is lower than for the proteome as a whole.

The intrinsic hypermutability of the antibody variable regions in B-cells is attributable to the behavior of activation-induced cytidine-deaminase (AID) and decays with increasing distance from the transcription initiation site (18, 19). The frequency of unique TCEMs shows a distinct trend from the N-terminal portion of the IGHV through CDR3 as the germline motifs decrease (Figure 4). Hypermutation happens one nucleotide at a time and mostly in hotspots within the CDRs. However, as we show in Figure 8, the impact on immunological recognition and function extends over a broader sequence span, as do the multiple selection pressures subsequently applied to the resultant variable region sequences. Considering a minimal 23-mer, as described, every amino acid has a role in 22 more overlaid peptide spans. Every mutation in one peptide has a Sudoku puzzle-like impact on the overlapping peptides. Within any given epitope peptide, the subdomains of cathepsin cleavage, GEM binding, and three overlaid TCEM registers each have different recognition rules, which in total provide a read-out of the immunologic function of that peptide. This pattern is repeated and superimposed for every sequential peptide across a protein.

The remarkable pattern of re-use of TCEMs observed in IGHV suggests a previously unrecognized dimension to the T-cell:pMHC-interaction. It also provides important clues to interactions that occur among T-cells, B-cells, and other APCs in the broader functioning of the immune system, which we discuss below.

### Polyspecificity

With only five variable amino acids of a peptide bound in a pMHC actually exposed to the T-cell, the maximum number of possible TCEM amino acid configurations in any of the registers is 20^5^ (3.2 × 10^6^). We show that the repertoire of TCEMs in the IGHV is actually much smaller, with some motifs used at very high frequency and others rarely. The high rate of re-use means that a relatively limited repertoire of cognate T-cell receptors can provide help to a diverse range of B-cell clonotypes that present the same motif in their pMHC. This is consistent with the calculations of Mason of the limited size of T-cell populations (20) and the observations of others of the polyspecificity of T-cell receptors (21–24).

Thinking beyond IGHV, how then can such a pattern of TCEM re-use also provide the necessary breadth of coverage and specificity to respond appropriately to all incoming antigens? Any particular TCEM can be present in one protein with a high affinity GEM, but occur in another protein with a low affinity GEM for the same MHC allele (as we show in Figure 7). By examination of several large random sets of non-immunoglobulin proteins we confirmed that the pMHC affinity for a peptide is uncorrelated with TCEM (not shown). In MHC class II bound peptides, the 10 amino acids in the GEM provide 20^10^ potential variants and the GEM of MHC class I provide 20^4^ variants (for 15-mer and 9-mer peptides, respectively). Hence by use of combinatorial TCEM × GEM motif recognition, the potential repertoire of pMHC class II complexes is expanded to ~10^19^ for each MHC class II allele, all while only needing to interact with up to 3.2 million unique T-cell clonotypes per MHC allele.

### Signal strength determines outcome

A number of different mathematical models have been derived for T-cell stimulation. Kinetic proofreading concepts form the backbone of many of the models where TCR:pMHC engagement triggers a series of signaling events governed by the engagement frequency and duration (25).

The dynamics of the pMHC:T-cell engagement is conditioned by three factors: (i) frequency of appearance of a cognate T-cell:TCEM pair; (ii) dwell time of the peptide in the MHC groove, determined by the GEM affinity; and (iii) on-off rate of T-cell receptor binding to the outwardly exposed surface of the pMHC, dependent on both MHC allele and TCEM. In our formulation, the frequency class of a TCEM determines the number of cognate T-cells, which will engage with a presenting cell. Both the GEM and TCEM affect the aggregate duration of engagement, each in the context of the specific MHC allele.

It has been recognized that outcome of a T-cell epitope interaction is driven by overall signal strength (26) and leads cytokine responses causing either clonal expansion and up-regulation, or a down-regulation and apoptosis of the corresponding cells. Our observations indicate that frequency class of TCEM, and hence the number of cognate T-cells, is potentially a very important factor in determining signal strength and outcome. Motifs that are very common, and associated with GEM of high affinity would tend to lead to down-regulation or suppression. Those which are uncommon and have a competitive but not excessively strong binding affinity would be more likely to up-regulate (26, 27) and provide an immunostimulatory Th response.

Any one B-cell, bearing many MHC molecules, can simultaneously present many copies of many different TCEM from its own IGHV. Potentially, this number is up to about 115 different variable region peptides if these also bind the MHC (i.e., peptides with any of about 130 index positions, −15, as the length of a MHC class II binding peptide we have elected). The peptides will compete for duration of MHC binding based on affinity. Also competing will be peptides from constant chains, which while having a null effect will occupy MHC. The most common TCEM motifs (FC1–3) provide a high probability of encountering cognate T-cells, which provide some T-cell help. The rarer TCEM allow fine tuning by providing more specific help. As we have shown, on average only three or four unique TCEM occur per IGHV molecule; these operate in the context of their more common neighbors. The outcome of help vs. suppression depends on the overall balance of TCEM presented by each cell. Rarer motifs which bind competitively will favor clonal expansion and selection and immunodominant epitopes are those that succeed in this process in the face of the competing immunosuppressive motifs.

Whether a specific peptide acts as a T-regulatory (Treg) epitope would be determined both by the frequency of the TCEM it contains and by its binding to host HLA. Personal history of epitope exposure will determine the particular array of TCEMs in an individual antibody repertoire (as opposed to the generic set we analyzed in our 40K set). This may explain why identical twins have differing T-cell responses and TCR repertoires (28). Furthermore, an individual’s IGHV repertoire can vary over time based on epitope exposure. It follows that Treg epitopes are both personal and dynamic. However, high frequency, broadly binding TCEMs may result in Tregs common to many individuals.

Major histocompatibility complex class I and II binding peptides commonly overlap, facilitating cross presentation (8). The overlap of the TCEM registers (I, IIa, and IIb) within the same peptide sequences (as shown in Figures 6 and 7), as well as host heterozygosity, means that the modulating effects of multiple registers combined can contribute further variability. Another dimension is that in order to be presented as a pMHC, the peptide must be appropriately excised by endopeptidases. As the cathepsin profile differs among classes of APC, the functional outcome may vary according to the presenting cell (10, 29).

### Mechanism for memory

Any SHM event that generates an immunoglobulin with a TCEM that engages a TCR and provides up-regulatory cytokine stimulation will lead to clonal expansion of T-cells bearing that TCR. The TCEM frequency patterns we show indicate that the same motifs are generated over and over again, so while mutation is stochastic, the cascade of T-cell clonal expansion events is not random but follows repeated pathways. The overlay of recognition signals (as shown in Figure 8) exerts a complex selective process determining, which motifs get presented. It has been widely recognized that tonic stimulation is necessary to maintain a repertoire of T-cells (30). T-cells binding more common TCEMs, the most polyspecific or public motifs (21), provide initial T-cell help and initiate clonal expansion, but is then joined by a more specific engagement initiated by more rare TCEMs. This pattern is seen in the frequency distribution pattern of IgM compared to IgG motifs.

The data presented show that peptides from endogenous immunoglobulins presented by B-cells and APCs can provide a balanced and constant source of TCEMs with a clear and maintained frequency distribution. This, under normal circumstances can provide homeostasis and a balanced repertoire, effectively a self-reference profile for the entire proteome. As new TCEM from novel exogenous antigen sources (e.g., an infection) are added, the balance may shift temporarily, with more T-cells engaging the newly presented TCEM, superimposed on the background frequency distribution. Troy and coworkers have shown how a novel clone competes for space to expand (31, 32). T-cell clones whose frequency or affinity make them uncompetitive, or which generate such a high signal strength as to be down-regulated will be eliminated to make space. As the novel antigen stimulus is removed following a primary response, the newly responding T-cell clone contracts, but some remain as a component of the new homeostasis (27, 33). On secondary exposure an anamnestic response is jump-started from these remaining T-cells from the initial response, which already bind more specific TCEM. This clonal-pattern imprinting also may explain heterologous immunity and original antigenic sin, in which exposure to a new antigen recognized to comprise a high frequency polyspecific TCEM may lead down a prior pathway of clonal expansion (34–36). Exposure to exogenous antigens is short-lived, but exposure to endogenous IGHV is on-going. Thus, exposure to a new antigen will tilt the repertoire toward novel TCEMs, but over time it will self-correct to the homeostatic IGHV frequency pattern.

Although this discussion focuses on endogenously produced immunoglobulins processed by both B-cells and APC, TCEM presentation can also arise from processing of exogenous immunoglobulins by dendritic cells and macrophages. This includes immunoglobulin received through maternal transfer (37) or by therapeutic administration, and may explain the immune re-balancing function of IVIG (38).

Endogenous immunoglobulins are the source of tonic stimulation of the T-cell population. Polyspecific cognate T cell clones will respond in numbers proportional to the specific frequency profile of TCEMs. This implies that memory can be maintained through polyspecific recognition, and anamnestic responses launched from within this cycling T-cell population. The propensity of any endogenous immunoglobulin variable region to maintain tonic stimulation of common TCEMs provides immediate responders, as well as an array of less common TCEMs, which can refine this response. The combined response is the basis for accelerated clonal maturation toward the specific recall response. Meanwhile the associated GEM binding provides combinatorial specificity. In this process, there will be subsets of T-cells and B-cells that acquire cellular signatures that are commonly detected experimentally and attributed to memory cells. However, in the model, we describe here there is no need for an individual memory cell to be long-lived to ensure a trained response, and no requirement for storage space for highly specific memory cells. Memory can be maintained by the homeostatic population of T-cells which is responding to (or learning from) the frequency distribution of motifs presented to it from the balanced endogenous source, predominantly turnover of immunoglobulin peptides, and intermittently from exogenous sources.

### Relation to prior observations

The dissection of multiple overlaid systems of motif recognition is made possible by a combination of bioinformatics, patterns deduced from structural biology, and the advances in sequencing technology that have produced large datasets of immunoglobulin variable regions. Teasing the motif recognition apart in an experimental format, compounded by overlapping peptides, heterozygosity, and multiple HLA loci is challenging. Our findings and the discussion of their implications are consistent with those of many prior investigators. Without engaging here in a full review of the extensive related literature on T-cell repertoires and B–T cell cooperation, we will very briefly identify a few diverse points, in addition to those already cited above, that the material we have presented helps to bring together.

Jerne’s idiotypic network theory recognized the immune system relies on an interacting network of lymphocytes which recognize immunoglobulin variable regions, but interpreted this as the production of anti-idiotypic antibodies and further antibodies thereto. He proposed this as an antibody-determined phenomenon based on the space–shape (idiotope) of the variable region (39). Cohen elaborated on this with a concept of a self-reference profile (40). We also attribute memory to a network. The patterns we present indicate a network in which the primary amino acid sequence of immunoglobulins is processed and presented to generate T-cell epitopes. The frequency distribution of the TCEM component of such epitopes maintains a self-reference profile, recognized in the context of the individual’s immunogenetics.

The role of B-cells as APCs has long been recognized (41), as is their presentation of endogenous antibody derived peptides (1). Bogen and Weiss proposed that MHC-restricted presentation of immunoglobulin variable region peptides to T-cells regulates the immune response, and also plays a role in affinity maturation and memory (17). We concur, and suggest that the potential contribution of such peptides is much greater than expected. The outcome, or signal strength, is then the balance of the common and the rare peptides.

Given the finite size of the T-cell pool, a regulatory mechanism to ensure that novel epitope driven clonal expansion does not obliterate the homeostatic repertoire is essential. The need for constant stimulation to maintain the T-cell repertoire has been understood and attributed to self-MHC ligands (42). Experience working with the therapeutic value of IVIG in reconstituting T-cell diversity led to the hypothesis put forward by Joao (43) that immunoglobulin is essential to maintain a diverse T-cell repertoire. The continued availability of immunoglobulin-derived peptides provides both a self-righting mechanism and on-going stimulation. At the same time, the distribution frequency we show, with much repetition of TCEMs, suggests that immunosuppression and/or tolerization is very common and that only uncommon TCEMs allow the response to rise above the immunosuppression.

Polyspecificity and heterologous immunity go hand-in-hand. Our demonstration of re-use of TCEMs implies that heterologous immunity is inevitable; the degree of cross-reactivity being modulated by the associated binding affinity (36). The consistent frequency distribution of TCEMs from IGHV provides a “self-reference profile” for the T-cell repertoire which, while it is dynamic and can “learn” based on new antigen exposure and antibody generation, reverts to a balanced composition because of the on-going turnover of endogenous antibodies.

## Conclusion

Our observations of the patterns of peptide motifs from endogenously generated immunoglobulin variable regions suggest a relatively simple, yet powerful means of coordination of T-cell polyspecificity with responsiveness to widely diverse epitopes. This mechanism is based on the combinatorial power of the overlay of short motifs of non-continuous amino acids, plus duration, and frequency of TCEM interaction. The same combination of signals may also modulate the outcome of T-cell engagement. The frequency profile of TCEMs in IGHV also points to an economical system for maintenance of homeostasis, memory, recall, and self-discrimination. The mechanisms we describe provide yet another example of the interdependence of B-cells and T-cells.

## Methods

### Database assembly

Approximately 45,000 heavy chain variable regions were retrieved from NCBI Protein resource with a search argument “(IGHV) and (*Homo sapiens*).” Numbers of IGHV greatly exceed those of light chain sequences. Because of the way proteins are deposited and annotated the heavy chain and light chain pairs are not explicitly connected. Therefore, only IGHV sequences were included in the current analysis. Search arguments were applied to eliminate sequences for which the GenPep metadata attached to the accession indicated association with an immunopathology (lymphoma, leukemia, lupus, rheumatoid arthritis, and multiple sclerosis). Manual curation was used to remove a small number of sequences that were obviously not immunoglobulins. Duplicate gi numbers were removed to make the data sets non-redundant. From the master set, a non-redundant subset of 2834 immunoglobulins was then extracted that was immunoglobulin class-defined. The remaining dataset included 39,982 non-class-defined immunoglobulins, not associated with immunopathology. This dataset, the “40K set” comprises many different accession groups from studies carried out over a considerable period of time so can be considered a representative sample of “natural” human immunoglobulins. We compared the frequency of germline-origin with the frequencies found by IGHV repertoire sequencing in healthy humans (44). The correlation coefficient was 0.95 between the two sets and thus the 40K set has close resemblance to a healthy human. Accessions with signal peptides were identified and signal peptides removed using the combined signal peptide and transmembrane predictor Phobius (phobius.sbc.su.se). All sequences longer than 130 amino acids were truncated at that point, consistent with the practice of IMGT. The approximate positions of the three CDRs have been indicated in Figure 2 relative to standard IGHV sequence landmarks. Genbank accession indices of the final 40K IGHV reference set are provided in Supplemental File 1.

The separate subset of 2834 class-defined IGHV IgG (*n* = 1630), IgE (*n* = 667), and IgM (*n* = 537) was derived similarly by adding additional key words to the search arguments. There are inevitable biases in the class-defined datasets. For example, the sources of nearly all of the IgE sequences were from cohorts of asthmatics (11–13) and either did not include or identify the sequences of non-asthmatics in the cohorts. Many of the class-defined IgG sequences were derived from an HIV study (45), however, subsequent analysis showed all TCEM in the IgG subset were also in the main 40K database. Germline IGHV (*n* = 161) were obtained from the IMGT repository (www.imgt.org), and IGHC class reference sequences from Genbank. The human proteome was downloaded from www.uniprot.org. The dataset comprises approximately 81,000 proteins including multiple isoforms of some proteins. This UniProt dataset includes immunoglobulin sequences; these were removed by manual curation.

For each of the analyses described below each sequence in the above databases was broken into 15-mers and 9-mers, each offset by a single amino acid. Thus, the combined set of 40,000 IGHV sequences resulted in approximately 4.2 × 10^6^ peptides. The same processing was carried out with the IGHV germline sequences, immunoglobulin constant regions, and the human proteome.

### TCEM classification

The determination of TCEM and GEM non-continuous peptides was derived from the work of Rudolph et al. (5) and Calis et al. (6) which cataloged the contact points of different T-cell receptor: pMHC structural models and characterized the atomic interactions between the amino acids in the pMHC and the TCR, as well as those involved in the binding of the peptide in the groove of the MHC. Relative to the binding pocket P1–P9 of a 9-mer for CD8 and the central 9-mer core of a 15-mer for CD4 T-cells, three different types of T-cell exposed pentamer motifs were deduced from the structural data. For CD4+ the predominant interactions of the T-cell receptor are approximately equally divided between those with the amino acids at the sequence positions 2,3,5,7,8 and at −1,3,5,7,8. In contrast for CD8+ receptor binding, the predominant interactions are with the continuous group 4,5,6,7,8 with 5 being by far the strongest. There is some plasticity in these discrete categories, but the predominant interactions can be deduced from the results tabulated by Rudolph et al. (5) and Calis et al. (6); these are tabulated in Table S3 in Supplementary Material and shown in Figure 1B. In the IGHV datasets approximately 30% of the total 2,3,5,7,8 and −1,3,5,7,8 are overlapped, hence the hexamer −1,2,3,5,7,8 contributes to binding. Pentamer TCEM sub-sequences were extracted from all possible 9-mer and 15-mer registers within the databases of immunoglobulins. Likewise the intercalated GEM sequences were extracted. With access to a larger structural database of TCR it may be possible to attribute relative weighting based on TCR peptide contact probability, but for the present analysis all the indicated pocket positions were given equal weights.

It should be noted that Rudolph et al. provide data for MHC class IA and IB and for MHC class II DR alleles, but not DP or DQ. Whether these alleles utilize the same contact arrangements of TCEM motifs as DR is not known. The approach we employ could be applied to these alleles and likely does not affect the overall outcome; indeed TCEM registers and recognition by the other MHC class II alleles by the TCR may offer additional specificity filters. By default we focused on 9-mer and 15-mer MHC binding peptides, but the same approach would be equally applicable to MHC binding peptides of other lengths.

### Predicted MHC binding affinity

For each derived 9-mer and 15-mer peptide, the predicted binding affinity to 37 MHC class I and 28 MHC class II alleles was determined using neural network regression equations that were developed using the allelic affinity (IC_50_) data retrieved from www.iedb.org. The background of the predictions using amino acid principal components has been published previously (46, 47). The predictions have now been improved and optimized, using new JMP^®^ software releases and the expanded peptide affinity training sets available at IEDB (as of June 2012). Training sets for 9-mers and 15-mers were used from the IEDB IC_50_ datasets. These are the most common lengths found experimentally for MHC class I and class II bound peptides. It is recognized that the MHC class I peptides can range from 8 to 11 amino acids and the MHC class II peptides can be slightly less than the 15 or extend to 20-mers. The training set sizes were used for the indexing windows in all analyzed sequences as affinity predictions for peptides outside the training size cannot be made.

In brief, ensembles of the neural network predictions were generated using a bootstrap aggregation (“bagging”) approach, where multiple random subsets of the peptide training sets were used independently to develop a neural network regression prediction equation for each allele using a 5-kfold cross validation process to estimate the log_e_ IC_50_. The result of one round of this process is a neural network prediction equation derived from a single random combinatorial subset of the data. The process was repeated with different random subsets a total of 300 times for each allele training set. Finally, ten ensembles with the best predictive performance, as judged by their training and validation statistics were chosen and used to estimate the mean log_e_ IC_50_. This approach enables the prediction not only of the mean, but also a standard deviation of the predictions of the ten ensemble sets. The standard deviation is a metric that provides a reliability estimate for the predictions that is meaningful to experimentalists. The overall standard deviation average for the all of the alleles predicted is ±0.7 log_e_ units. The range in this metric varies for the different alleles from 0.5 to 2.3 log_e_ units and is traceable to a characteristic of the training sets themselves. The value of this variance metric obtained for an allele with the training set peptides is highly correlated with the metric for the same allele in other proteins (Bremel, unpublished data).

Within any protein, binding to the different alleles exhibits different distributional characteristics, making comparisons among alleles difficult. Thus, in a separate computation, all log_e_ IC_50_ predictions from the neural network predictions for each allele are also standardized to zero mean and unit variance within each protein using the Johnson Sb standardization platform of JMP^®^. This is a distribution transformation known to be robust for distributions with various degrees of skewness and kurtosis and converts the raw affinity data for each of the alleles to a Gaussian distribution with a mean of zero and a SD of 1. After transformation all alleles are in a common scale and thus statistical analysis can be done without concern for scale-effects.

The alleles for which predicted binding affinity determinations were made are DRB1*01:01, DRB1*03:01, DRB1*04:01, DRB1*04:04, DRB1*04:05, DRB1*07:01, DRB1*08:02, DRB1* 09:01, DRB1*11:01, DRB1*12:01, DRB1*13:02, DRB1*15:01, DRB3*01:01, DRB3*02:02, DRB4*01:01, DRB5*01:01, DPA1*01:03-DPB1*02:01, DPA1*01:03-DPB1*04:02, DPA1*01:03-DPB1*04:01, DPA1*02:01-DPB1*01:01, DPA1*02:01-DPB1*05:01, DPA1*03:01-DPB1*04:02, DQA1*01:01-DQB1*05:01, DQA1*01:02-DQB1*06:02, DQA1*03:01-DQB1*03:02, DQA1*04:01-DQB1*04:02, DQA1*05:01-DQB1*02:01, DQA1*05:01-DQB1*03:01, A*01:01, A*02:01, A*02:02, A*02:03, A*02:06, A*03:01, A*11:01, A*23:01, A*24:02, A*24:03, A*26:01, A*29:02, A*30:01, A*30:02, A*31:01, A*32:01, A*33:01, A*68:01, A*68:02, A*69:01, B*07:02, B*08:01, B*15:01, B*15:03, B*18:01, B*27:05, B*35:01, B*40:01, B*40:02, B*44:02, B*44:01, B*51:01, B*53:01, B*54:01, B*57:01, B*58:01.

### Predicted endosomal cathepsin cleavage probability

The probability of cleavage of each protein by human cathepsin B, L, or S was determined for proteins based on successive octomers indexed by a single amino acid throughout the primary amino acid sequence. As for binding affinity the cleavage predictions were accomplished using previously described methods by neural network predictors based on principal component analysis of amino acid physical properties (6, 46, 47). A bagging process, as described above for the affinity predictions, was also used for prediction of cathepsin cleavage probability using the “neural” platform of JMP^®^. A probability of cleavage (scaled 0–1) is computed for the central dipeptide of an octomer. This dipeptide by convention is called P1P1′ and the scissile bond cleaved by the peptidase occurs between these two amino acids. A large proteomic data set consisting of cleavages of the three indicated cathepsins obtained at different pHs and at different time intervals was used for training the neural networks (48). Ensembles of prediction equations were created for each different P1P1′ dipeptide combination in the training sets for each peptidase. The discriminant neural network ensembles that result from this process separately and simultaneously predict the cleavage and non-cleavage probability. The final metric for each dipeptide pair in a protein molecule is the median of the cleavage predictions of all of the ensembles. The overall sensitivity and specificity of the prediction equations indicated by the AROC is 0.83 and differs for each P1P1′ and with a range from 0.71 to 0.93. This type of prediction attempts to reduce enzyme reactions occurring in a complex endosomal milieus to a binary result. Nevertheless, when these equations were tested against several mass spectrometry datasets of CLIP (49) and self-peptides (50) they were found to produce cleavage prediction results consistent experimentally determined peptides in the datasets (Bremel and Homan, unpublished).

All sequence manipulations and statistics reported were carried out with JMP^®^ 11 (SAS Institute, Cary, NC, USA).

## Conflict of Interest Statement

Both authors are employees and equity holders in ioGenetics LLC, the parent company of EigenBio LLC. Aspects of the work described are the subject of patent filings.

## Supplementary Material

The Supplementary Material for this article can be found online at http://www.frontiersin.org/Journal/10.3389/fimmu.2014.00541/abstract

## Abbreviations

APC, antigen presenting cell; CDR, complementarity determining regions; CLIP, class II-associated invariant chain peptide; CSO, cleavage site octomer; FC, frequency classification; FW, Framework region; GEM, groove-exposed motif; IGHC, immunoglobulin heavy chain constant region; IGHV, immunoglobulin heavy chain variable region; IMGT, the international ImMunoGeneTics information system^®^ http://www.imgt.org; IVIG, intravenous immunoglobulin; MHC, major histocompatibility complex; pMHC, peptide MHC complex; SHM, somatically hypermutated; TCEM, T-cell exposed motif; TCR, T-cell receptor; Th, T helper.
