# Supplementary material for: Frequency Patterns of T-Cell Exposed Amino Acid Motifs in Immunoglobulin Heavy Chain Peptides Presented by MHCs
Source: Front Immunol. 2014 Oct 28;5:541. doi: 10.3389/fimmu.2014.00541 (PMC4211557; doi:10.3389/fimmu.2014.00541)
Supplement: Supplementary file 1 [file Data_Sheet1.ZIP › Supplementary Tables and Figures.pdf]

Supplementary Material

Frequency patterns of T-cell exposed motifs in immunoglobulin heavy chain peptides presented by MHCs.

Robert D. Bremel[1]*, E. Jane Homan[1].

[1]EigenBio LLC, Madison, WI, USA

*** Correspondence:** Robert D Bremel, EigenBio LLC, 3491 Anderson Street, Madison WI, 53704 USA

robert_bremel@eigenbio.com

Supplementary Figures and Tables

Supplemental Table 1:

T-cell exposed motif frequencies in the IGHV. The frequency class (FC) is computed as the $-\log_2$ (frequency) where the frequency represents the number of times that a particular motif occurs in the database of 40,000 IGHV sequences. The "Germline" motifs are pentamers that are identical to that found in at least one of the human IGHV germlines and the "Mutated" sequences are pentamers generated by somatic hypermutation. The "unweighted" columns represent the number of unique motifs that are found in each FC and the weighted columns are frequency weighted an represent the number of times that these motifs are found in the different IGHV sequences and were used to compute the "per molecule" occurrence of each type of FC. A value of 106 amino acids per IGHV is the median size of the molecules in the database once the signal peptides are removed.

TCEM IIa 2,3,5,7,8

FC	Count Germline unweighted	Count Mutated unweighted	Probability Germline unweighted	Probability Mutated unweighted	Count Germline weighted	Count Mutated weighted	Probability Germline weighted	Probability Mutated weighted	# per molecule (106 aa) Germline	# per molecule (106 aa) Mutated	per molecule total
1	10	2	0.005	0.000	247,457	52,173	0.100	0.027	6.011	1.267	7.278
2	50	3	0.024	0.000	756,946	38,853	0.307	0.020	18.387	0.944	19.330
3	37	8	0.018	0.000	243,346	47,642	0.099	0.025	5.911	1.157	7.068
4	118	15	0.057	0.000	431,804	50,382	0.175	0.027	10.489	1.224	11.713
5	153	66	0.073	0.000	260,003	108,256	0.106	0.057	6.316	2.630	8.945
6	323	147	0.155	0.001	288,017	130,136	0.117	0.068	6.996	3.161	10.157
7	285	265	0.137	0.001	128,884	113,396	0.052	0.060	3.131	2.754	5.885
8	281	844	0.135	0.003	62,998	181,580	0.026	0.096	1.530	4.411	5.941
9	250	1,532	0.120	0.006	28,949	167,360	0.012	0.088	0.703	4.065	4.768
10	159	3,030	0.076	0.011	9,459	165,728	0.004	0.087	0.230	4.026	4.255
11	114	6,118	0.055	0.022	3,247	166,073	0.001	0.087	0.079	4.034	4.113
12	133	12,226	0.064	0.045	1,860	163,784	0.001	0.086	0.045	3.978	4.024

13	72	23,794	0.035	0.087	493	153,740	0.000	0.081	0.012	3.734	3.746
14	46	33,994	0.022	0.125	152	114,829	0.000	0.060	0.004	2.789	2.793
15	30	55,234	0.014	0.202	60	110,468	0.000	0.058	0.001	2.683	2.685
16	23	135,739	0.011	0.497	23	135,739	0.000	0.071	0.001	3.297	3.298
Total	2,084	273,017	1	1	2,463,698	1,900,139	1	1	59.845	46.155	106

TCEM IIb -1,3,5,7,8

FC	Count Germline unweighted	Count Mutated unweighted	Probability Germline unweighted	Probability Mutated unweighted	Count Germline weighted	Count Mutated weighted	Probability Germline weighted	Probability Mutated weighted	# per molecule (106 aa) Germline	# per molecule (106 aa) Mutated	per molecule total
1	8		0.004		205,157		0.083		4.983		4.983
2	52	4	0.025	0.000	786,946	55,548	0.319	0.029	19.115	1.349	20.465
3	34	6	0.016	0.000	221,112	36,763	0.090	0.019	5.371	0.893	6.264
4	124	15	0.059	0.000	457,559	53,220	0.185	0.028	11.114	1.293	12.407
5	146	68	0.069	0.000	252,228	111,763	0.102	0.059	6.127	2.715	8.842
6	341	154	0.162	0.001	309,417	136,101	0.125	0.072	7.516	3.306	10.822
7	288	277	0.137	0.001	129,852	118,813	0.053	0.063	3.154	2.886	6.040
8	277	871	0.132	0.003	62,150	187,031	0.025	0.099	1.510	4.543	6.053
9	237	1,601	0.113	0.006	27,326	174,743	0.011	0.092	0.664	4.245	4.908
10	160	3,005	0.076	0.011	9,657	163,955	0.004	0.086	0.235	3.983	4.217
11	130	6,262	0.062	0.023	3,782	170,573	0.002	0.090	0.092	4.143	4.235
12	134	12,420	0.064	0.045	1,885	166,187	0.001	0.088	0.046	4.037	4.083
13	66	24,267	0.031	0.088	453	156,524	0.000	0.083	0.011	3.802	3.813
14	46	34,562	0.022	0.125	162	116,736	0.000	0.062	0.004	2.836	2.840
15	38	55,576	0.018	0.201	76	111,152	0.000	0.059	0.002	2.700	2.702
16	20	136,946	0.010	0.496	20	136,946	0.000	0.072	0.000	3.326	3.327
Total	2,101	276,034	1	1	2,467,782	1,896,055	1	1	59.944	46.056	106

TCEM I 4,5,6,7,8

FC	Count Germline unweighted	Count Mutated unweighted	Probability Germline unweighted	Probability Mutated unweighted	Count Germline weighted	Count Mutated weighted	Probability Germline weighted	Probability Mutated weighted	# per molecule (106 aa) Germline	# per molecule (106 aa) Mutated	per molecule total
1	14	4	0.007	0.000	334,276	90,480	0.142	0.045	8.150	2.206	10.356
2	48	2	0.025	0.000	708,118	33,251	0.301	0.017	17.265	0.811	18.076
3	40	11	0.021	0.000	251,286	76,254	0.107	0.038	6.127	1.859	7.986
4	89	32	0.046	0.000	327,065	117,167	0.139	0.059	7.974	2.857	10.831
5	133	92	0.069	0.000	230,305	154,339	0.098	0.077	5.615	3.763	9.378
6	318	168	0.166	0.001	284,778	144,900	0.121	0.073	6.943	3.533	10.476
7	253	238	0.132	0.001	111,240	101,016	0.047	0.051	2.712	2.463	5.175
8	276	798	0.144	0.003	62,791	169,550	0.027	0.085	1.531	4.134	5.665
9	240	1,329	0.125	0.005	27,570	145,059	0.012	0.073	0.672	3.537	4.209
10	138	2,725	0.072	0.010	8,196	148,655	0.003	0.074	0.200	3.624	3.824
11	95	5,821	0.050	0.021	2,716	158,779	0.001	0.080	0.066	3.871	3.938
12	123	11,247	0.064	0.041	1,661	150,004	0.001	0.075	0.040	3.657	3.698

13	69	22,453	0.036	0.082	465	144,637	0.000	0.072	0.011	3.527	3.538
14	39	32,951	0.020	0.120	133	111,216	0.000	0.056	0.003	2.712	2.715
15	30	54,220	0.016	0.197	60	108,440	0.000	0.054	0.001	2.644	2.645
16	14	143,085	0.007	0.520	14	143,085	0.000	0.072	0.000	3.489	3.489
	1,919	275,176	1	1	2,350,674	1,996,832	1	1	57.314	48.686	106

Supplemental Table 2:

TCEM motifs in heavy chain constant regions also found in the heavy chain variable region 40K database. Sequences were analyzed starting from the cysteine at position 25-27 of CH1 to accommodate differences in the length of the VDJ region.

	TCEM I			TCEM IIa			TCEM IIb		
Class	HC	# in IGHV database	%	HC	# in IGHV database	%	HC	# in IGHV database	%
IgA	294	18	6.12	282	29	10.28	286	25	8.74
IgD	334	21	6.28	335	22	6.56	331	26	7.85
IgE	363	36	9.91	361	39	10.80	366	33	9.01
IgG1	294	7	2.38	296	3	1.01	294	7	2.38
IgG2	294	8	2.72	287	8	2.78	290	7	2.41
IgG3	290	44	15.17	286	45	15.73	289	45	15.57
IgG4	288	10	3.47	282	12	4.25	290	8	2.75
IgM	399	27	6.76	410	14	3.41	411	9	2.18

Supplemental Table 3

Number of atomic contacts between the T-cell receptor and the peptide in the pMHC as tabulated from Rudolph *et al* (5). Two different MHC class II categories are used because the contacts appear in the tabulation to favor one or the other. In the IGHV database one-third of the motifs have a shared TCEM IIa and TCEM IIb configuration.

Pocket position	MHC II	MHC I
-4	5	
-3	14	
-2	7	
-1	29	
1	0	10
2	40	4
3	26	4
4	1	126
5	39	311
6	5	48
7	23	77
8	34	66
9	1	0
10	1	
11	0	
12	0	

Supplemental Figure S1

Distributions of predicted binding affinities of peptides in the IGHV for different IGHV families. The data, colors, and the orientation are the same as seen if Figure 2 A-D of the main text. The shaded areas are the approximate locations of the CDRs. Because of computational limits a subset of approximately one third of the 40K database are depicted. The number in parenthesis represents the number of IGHV in each of the figures. IGHV1 (2,276), IGHV2 (148), IGHV3 (9014), IGHV4 (1391), IGHV5 (243), IGHV6 (922), IGHV7 (32).

Supplemental Figure S2

Predicted binding affinities of peptides in the 161 germlines combined. The same data, colors and orientation are used as in Figure 2 (A-D) in the main text and in Figure S1. The approximate location of two CDR regions are shown as CDR3 is absent from the germlines.

Supplemental Figure S3

Predicted probability of cleavage of the IGHV by three endosomal cathepsins; cathepsin B, cathepsin L and cathepsin S. Cleavage predictions are made using the discriminate equations of a binary classifier neural network ensembles derived from large proteomic data sets and described in Bremel and Homan (7). The top 3 panels are for the combined 161 germline sequences and the bottom three panels are for the IGHV3 in the subset shown in Supplemental Figure 1. Only cleavage probabilities of > 0.5 are shown. The heavy bar depicts probability limits for the 25- and 75-percentile and the extensions mark the 10- and 90-percentiles. The gray shaded areas depict the approximate location of the CDRs. The cleavage predictions are made for the central dimer of an octomer.

Supplemental Figure S4

Positional frequencies of unique T-cell exposed motifs are shown for TCEM IIa, TCEM IIb, and TCEM I for IGHV3. The center of each of the TCEM are plotted against the $\log_2$ of the number of different motifs at the particular location in the IGHV. The upper panel shows IGHV3 germline sequences and the lower panel is from the entire SHM 40K data set that has 22,458 different IGHV3 proteins. The coloration is as described in Figure 2 of the main text.

Supplemental Figure S5

Positional frequencies for TCEM IIa in different germline families. Upper panels are germline sequences, lower panels are from the entire SHM 40K data set Description is the same as Figure 2 in the main text and Supplemental Figure S4.

Supplemental Figure S6

DRB allele affinity distribution histograms of the 351 peptides where YA~S~KG is found. Corresponds to the standardized distribution found in Figure __

Shown here is the $\log_e$ of the IC_{50} of the peptides predicted by the neural network ensembles. The overall mean and standard deviation are predicted for each peptide with ensembles trained with a bagging (bootstrap aggregation) process. The midpoint of the highest bar for DRB1*01:01 (4.75) corresponds to and IC_{50} of 116 nM and the overall range for the allele in different GEM contexts is from about 10 to 1000 nM.

Supplemental Figure S7

A. Venn diagram of the TCEM IIa (2,3,5,7,8) motifs in the entire, redundant, human proteome joined with the TCEM IIa (2,3,5,7,8) motifs in the 40K IGHV dataset. A total of 14.6% of the TCEMs frames found in the proteome match TCEM frames in IGHV. 85.5% of the unique motifs in the IGHV set are found in the proteome. In addition, the IGHV regions have a significant number of motifs not present in the proteome (14.5%). Presumably, these would be available potentially to provide T-cell match motifs from pathogens and other non-self antigens.

B. Relationship between TCEM frequency (-$\log_2$) of the IGHV and the human proteome. Bivariate modal clustering to produce spectral color contours at 5 percentile intervals. Borders are non-frequency-weighted histograms of the variables in the proteome.

Supplemental File 1

Text file of GI numbers of IGHV in the 40K dataset.

Figure S1

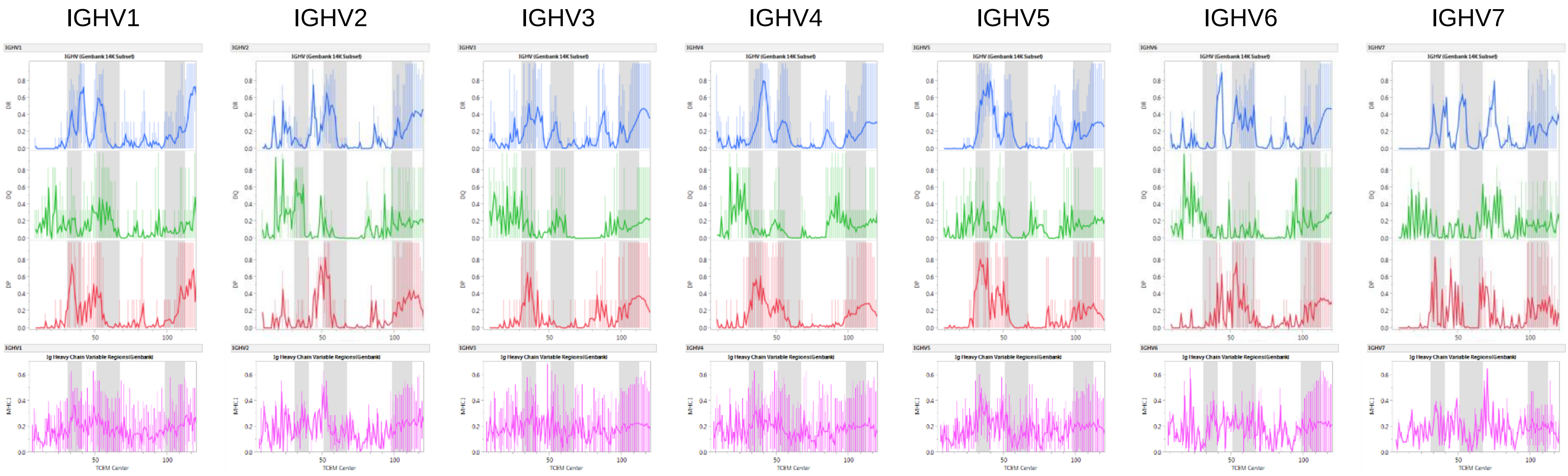
IGHV1
IGHV2
IGHV3
IGHV4
IGHV5
IGHV6
IGHV7
IGHV (Genbank 14K Subset)
Ig Heavy Chain Variable Regions(Genbank)
DR
DQ
DP
MHC I
TCEM Center

Figure S2

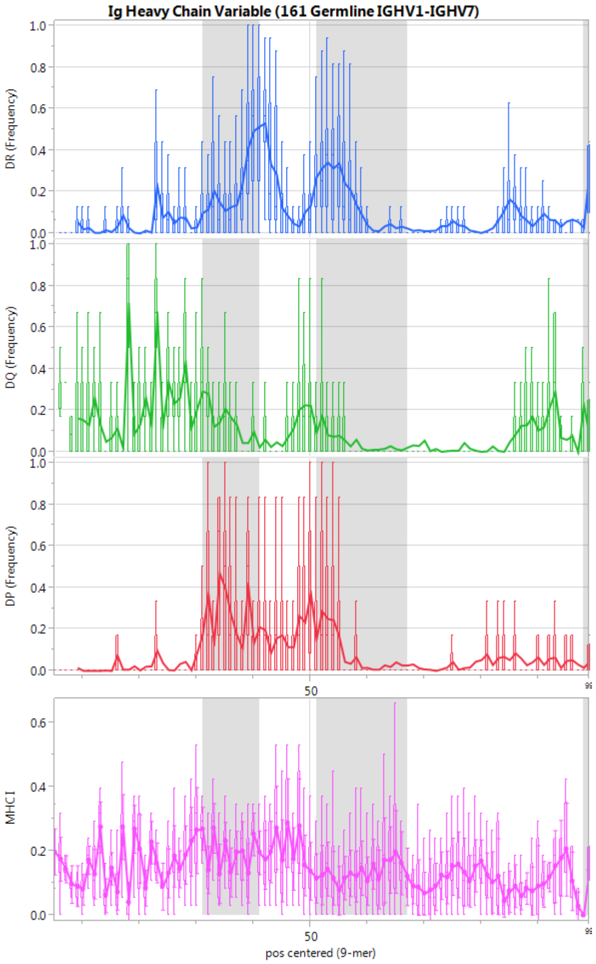

Ig Heavy Chain Variable (161 Germline IGHV1-IGHV7)
DR (Frequency)
DQ (Frequency)
DP (Frequency)
MHC I
1.0
0.8
0.6
0.4
0.2
0.0
50
pos centered (9-mer)

Figure S3

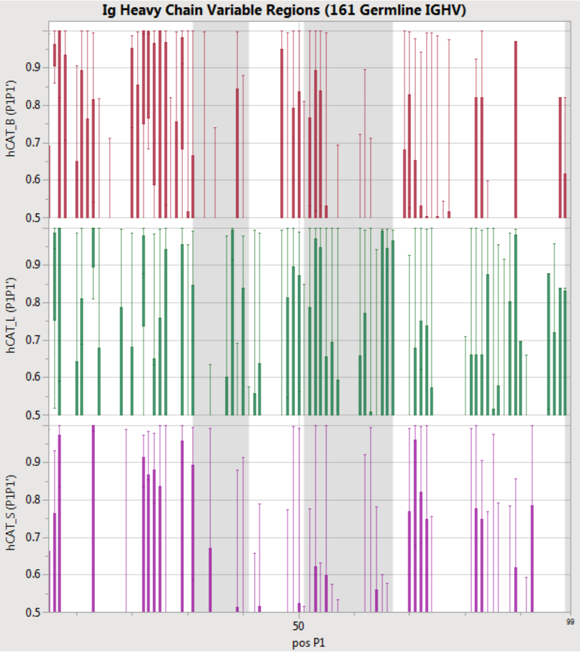
Ig Heavy Chain Variable Regions (161 Germline IGHV)
hCAT_B (P1P1')
hCAT_L (P1P1')
hCAT_S (P1P1')
0.5
0.6
0.7
0.8
0.9
50
99
pos P1

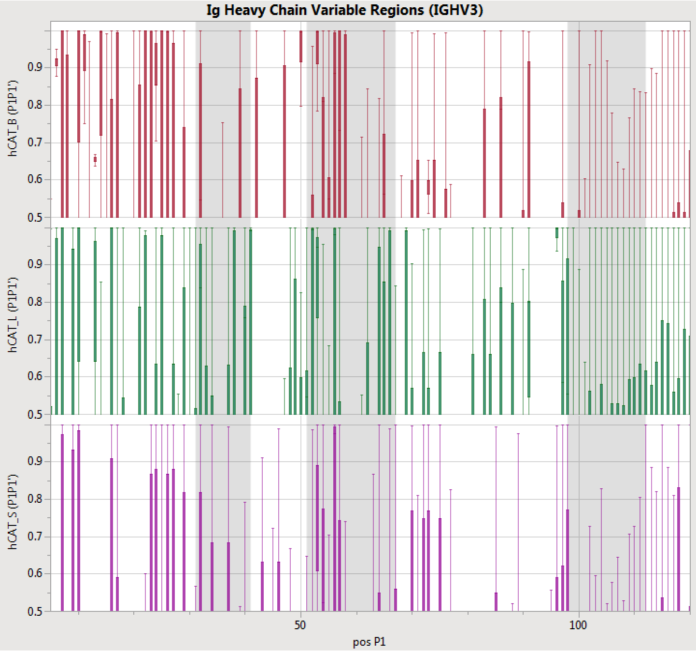
Ig Heavy Chain Variable Regions (IGHV3)
hCAT_B (P1P1')
hCAT_L (P1P1')
hCAT_S (P1P1')
0.5
0.6
0.7
0.8
0.9
50
100
pos P1

Figure S4

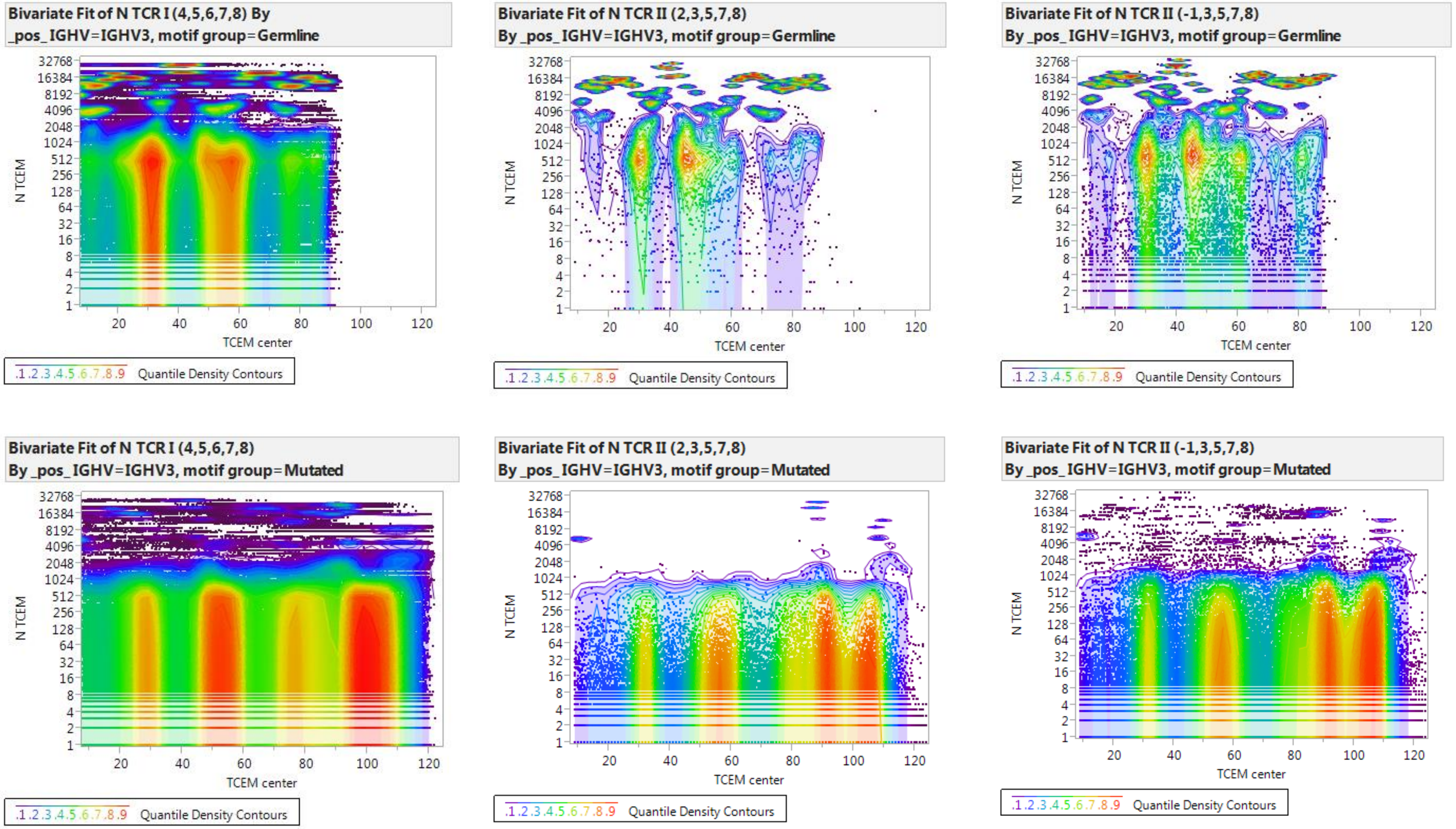
Bivariate Fit of N TCR I (4,5,6,7,8) By _pos_IGHV=IGHV3, motif group=Germline
Bivariate Fit of N TCR II (2,3,5,7,8) By _pos_IGHV=IGHV3, motif group=Germline
Bivariate Fit of N TCR II (-1,3,5,7,8) By _pos_IGHV=IGHV3, motif group=Germline
Bivariate Fit of N TCR I (4,5,6,7,8) By _pos_IGHV=IGHV3, motif group=Mutated
Bivariate Fit of N TCR II (2,3,5,7,8) By _pos_IGHV=IGHV3, motif group=Mutated
Bivariate Fit of N TCR II (-1,3,5,7,8) By _pos_IGHV=IGHV3, motif group=Mutated
N TCEM
32768
16384
8192
4096
2048
1024
512
256
128
64
32
16
8
4
2
1
TCEM center
20
40
60
80
100
120
.1 .2 .3 .4 .5 .6 .7 .8 .9 Quantile Density Contours

Figure S5

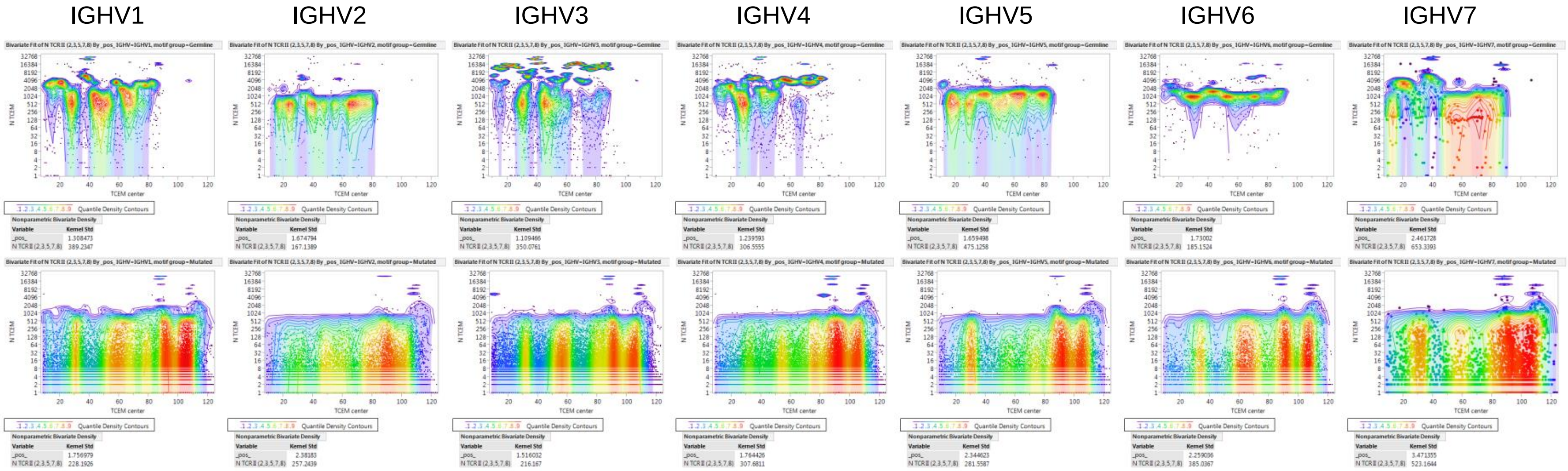

Figure S6

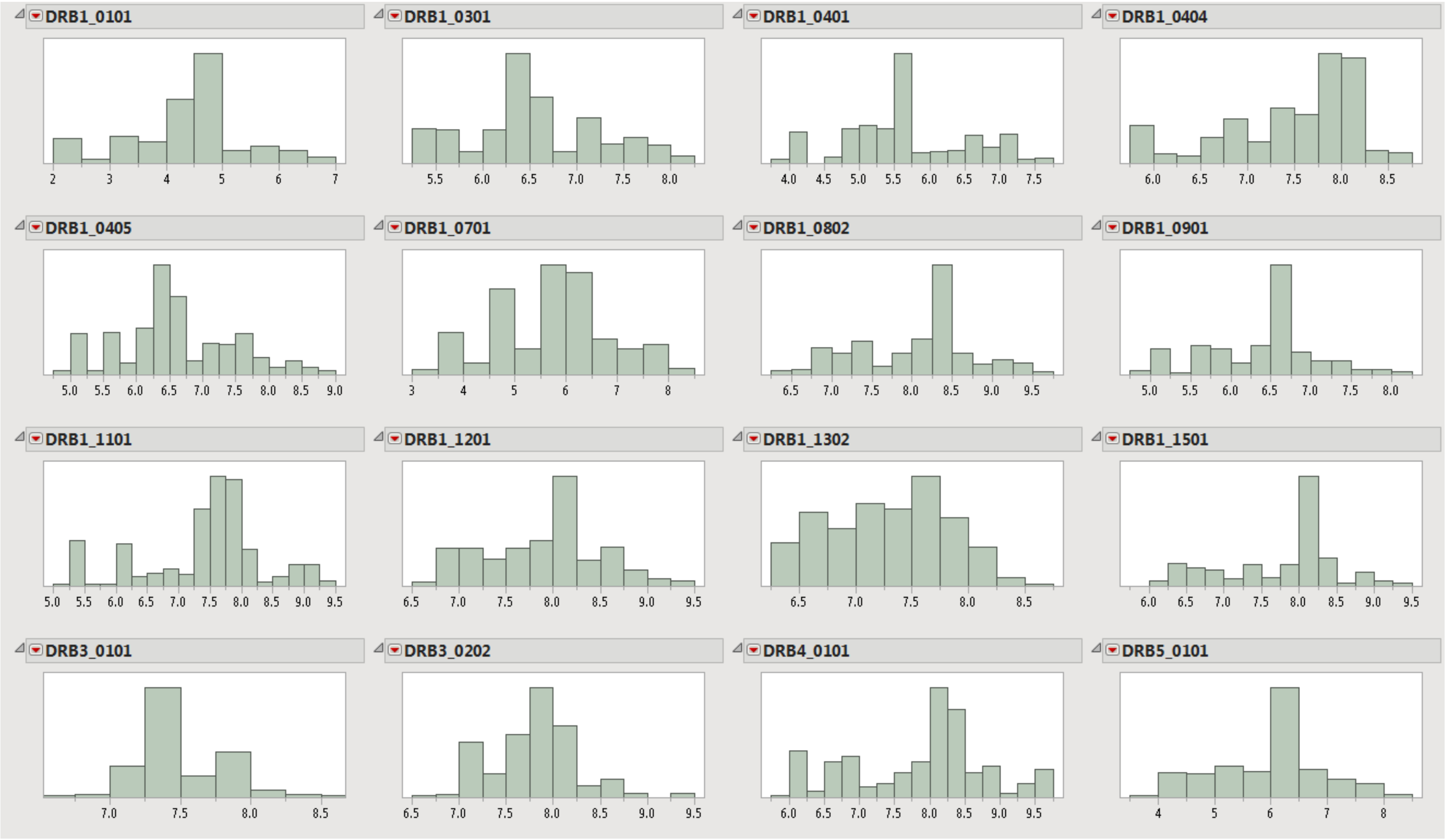
DRB1_0101
2 3 4 5 6 7
DRB1_0301
5.5 6.0 6.5 7.0 7.5 8.0
DRB1_0401
4.0 4.5 5.0 5.5 6.0 6.5 7.0 7.5
DRB1_0404
6.0 6.5 7.0 7.5 8.0 8.5
DRB1_0405
5.0 5.5 6.0 6.5 7.0 7.5 8.0 8.5 9.0
DRB1_0701
3 4 5 6 7 8
DRB1_0802
6.5 7.0 7.5 8.0 8.5 9.0 9.5
DRB1_0901
5.0 5.5 6.0 6.5 7.0 7.5 8.0
DRB1_1101
5.0 5.5 6.0 6.5 7.0 7.5 8.0 8.5 9.0 9.5
DRB1_1201
6.5 7.0 7.5 8.0 8.5 9.0 9.5
DRB1_1302
6.5 7.0 7.5 8.0 8.5
DRB1_1501
6.0 6.5 7.0 7.5 8.0 8.5 9.0 9.5
DRB3_0101
7.0 7.5 8.0 8.5
DRB3_0202
6.5 7.0 7.5 8.0 8.5 9.0 9.5
DRB4_0101
6.0 6.5 7.0 7.5 8.0 8.5 9.0 9.5
DRB5_0101
4 5 6 7 8

Figure S7

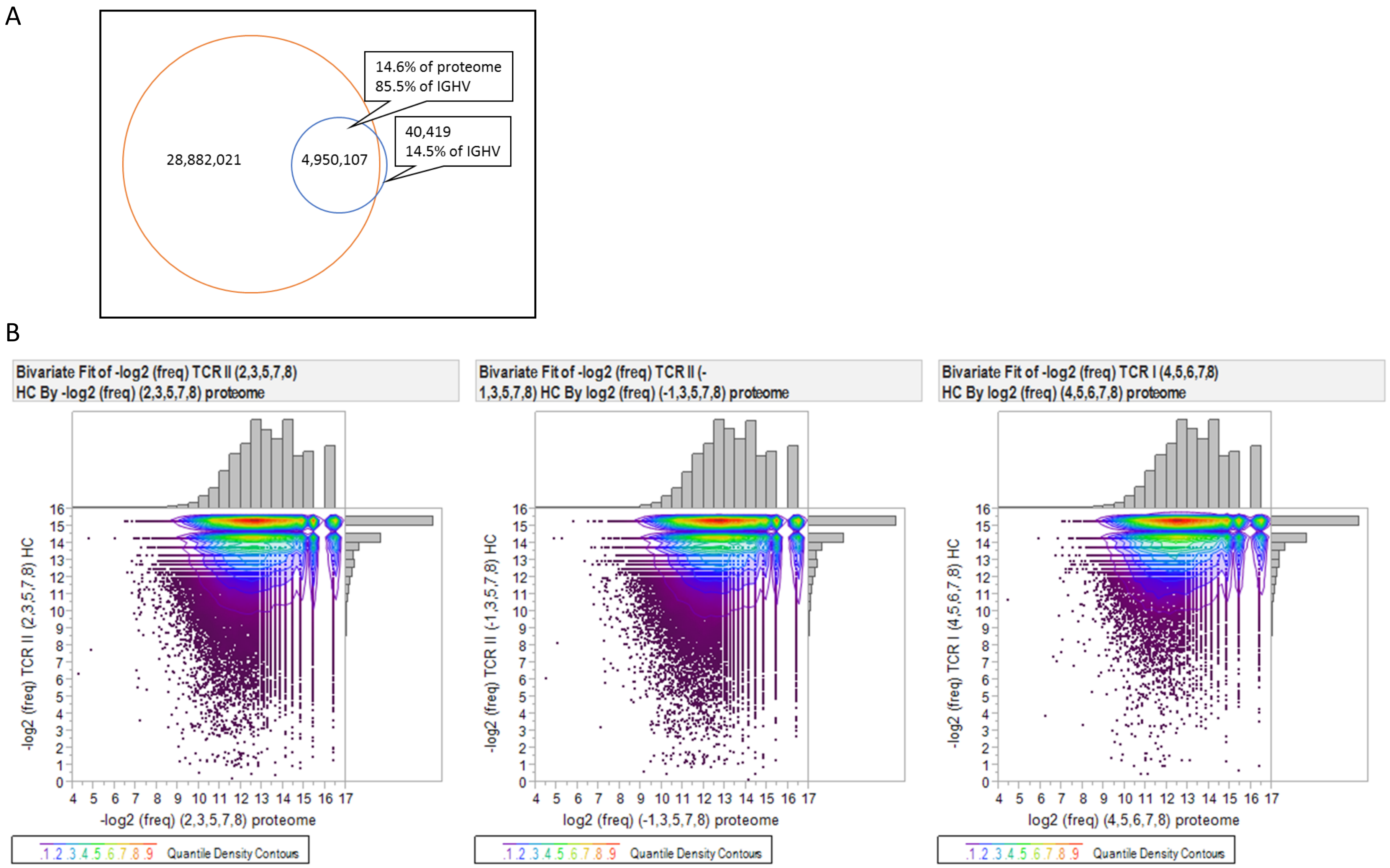
A
14.6% of proteome
85.5% of IGHV
40,419
14.5% of IGHV
28,882,021
4,950,107
B
Bivariate Fit of -log2 (freq) TCR II (2,3,5,7,8) HC By -log2 (freq) (2,3,5,7,8) proteome
-log2 (freq) TCR II (2,3,5,7,8) HC
-log2 (freq) (2,3,5,7,8) proteome
Quantile Density Contours
Bivariate Fit of -log2 (freq) TCR II (-1,3,5,7,8) HC By log2 (freq) (-1,3,5,7,8) proteome
-log2 (freq) TCR II (-1,3,5,7,8) HC
log2 (freq) (-1,3,5,7,8) proteome
Quantile Density Contours
Bivariate Fit of -log2 (freq) TCR I (4,5,6,7,8) HC By log2 (freq) (4,5,6,7,8) proteome
-log2 (freq) TCR I (4,5,6,7,8) HC
log2 (freq) (4,5,6,7,8) proteome
Quantile Density Contours
